# Structure‐Guided Design of a Group B *Streptococcus* Type III Synthetic Glycan–Conjugate Vaccine

**DOI:** 10.1002/chem.202000284

**Published:** 2020-04-01

**Authors:** Davide Oldrini, Linda del Bino, Ana Arda, Filippo Carboni, Pedro Henriques, Francesca Angiolini, Jon I. Quintana, Ilaria Calloni, Maria R. Romano, Francesco Berti, Jesus Jimenez‐Barbero, Immaculada Margarit, Roberto Adamo

**Affiliations:** ^1^ Research Center GlaxoSmithKline Plc Via Fiorentina 1 53100 Siena Italy; ^2^ Chemical Glycobiology Lab, CIC bioGUNE Basque Research Technology Alliance (BRTA) Bizkaia Technology Park 48160 Derio Spain; ^3^ Ikerbasque Basque Foundation for Science 48013 Bilbao Bizkaia Spain; ^4^ Department Organic Chemistry II University of the Basque Country UPV/EHU 48940 Leioa Bizkaia Spain

**Keywords:** carbohydrates, glycoconjugates, immunology, streptococcus, structural glycobiology, vaccines

## Abstract

Identification of glycan functional epitopes is of paramount importance for rational design of glycoconjugate vaccines. We recently mapped the structural epitope of the capsular polysaccharide from type III Group B *Streptococcus* (GBSIII), a major cause of invasive disease in newborns, by using a dimer fragment (composed of two pentasaccharide repeating units) obtained by depolymerization complexed with a protective mAb. Although reported data had suggested a highly complex epitope contained in a helical structure composed of more than four repeating units, we showed that such dimer conjugated to a carrier protein with a proper glycosylation degree elicited functional antibodies comparably to the full‐length conjugated polysaccharide. Here, starting from the X‐ray crystallographic structure of the polysaccharide fragment–mAb complex, we synthesized a hexasaccharide comprising exclusively the relevant positions involved in binding. Combining competitive surface plasmon resonance and saturation transfer difference NMR spectroscopy as well as in‐silico modeling, we demonstrated that this synthetic glycan was recognized by the mAb similarly to the dimer. The hexasaccharide conjugated to CRM_197_, a mutant of diphtheria toxin, elicited a robust functional immune response that was not inferior to the polysaccharide conjugate, indicating that it may suffice as a vaccine antigen. This is the first evidence of an X‐ray crystallography‐guided design of a synthetic carbohydrate‐based conjugate vaccine.

## Graphical abstract

**Deciphering the sugar glycocode**: Starting from the X‐ray crystallographic structure of a fragment from Group B *Streptococcus* type III capsular polysaccharide in complex with a protective mAb complex, a hexasaccharide comprising exclusively the relevant positions involved in interactions was obtained. By structural and immunogenicity studies it was shown that the synthetic hexasaccharide recognized the mAb similarly to the polysaccharide fragment and, conjugated to a carrier protein, suffices as vaccine antigen.

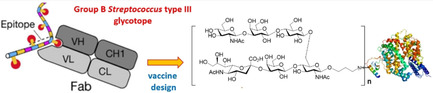

## Introduction

Over the latest years, structural vaccinology has come out as an emerging approach applied to modern protein‐based vaccines.^1^ Recent progress in synthetic carbohydrate chemistry, bioinformatics and analysis of carbohydrate–protein interactions facilitates the 3D‐structural elucidation of glycan–antibody complexes. Consequently, structural vaccinology principles can now be applied to carbohydrates, revealing the minimal glycan epitope (*glycotope*) suitable for elicitation of effective immune responses.^2^

Generally, polysaccharide structural antigenic determinants are composed of short and defined glycans varying in length from two to three monosaccharides as for β‐(1→2) mannans of the *Candida albicans* 3 cell wall, *Vibrio cholerae* O1,^4^ *Shigella flexneri* variant Y,^5^ and *Salmonella* 6 O‐antigens, up to nine sugar residues, as in the case of *S. flexneri* serotype 2a O‐antigen.^7^ The notion of minimal antigenic determinant and minimal immunogenic epitope is often not exchangeable, and typically fragments longer than the identified epitope are used as immunogens: for instance, a conjugated hexasaccharide from *V. cholerae* O‐antigen has been shown to be needed for immunogenicity,^8^ whereas a synthetic carbohydrate based vaccine prepared from a pentadecasaccharide of *S. flexeneri* 2a has been recently tested in human trials.^7^ Recent advancements in carbohydrate synthesis combined with structural glycobiology techniques such as Saturation Transfer Difference NMR (STD NMR), Surface Plasmon Resonance (SPR) and Glycoarray have enabled rapid identification of small glycan antigens to be used as immunogens in the design of vaccines against *Streptococcus pneumoniae*,^9^ *Burkholderia pseudomallei*,^10^ and *Clostridium difficile*.^11^

In contrast to those examples, the Group B *Streptococcus* (GBS) type III capsular polysaccharide (PS) has been proposed as prototype of a unique length‐dependent complex conformational epitope. GBS is a leading cause of bacterial sepsis and meningitis in neonates.^12^ Recently, GBS associated antimicrobial resistance^13^ has also emerged. Clindamycin‐resistant strains have caused more than 40 % of GBS infections, limiting prevention and treatment options for people with severe penicillin allergy in the US,^14^ and a human GBS ST1 isolate showed a surprisingly high penicillin resistance in Colombia.^15^

GBS PSs are constituted by multiple repeating units (RUs) (varying from ca. 50 up to ca. 300 per polymer) composed of four to seven monosaccharides shaped to form a backbone, to which one or two side chains are linked. Ten serotypes presenting a unique pattern of glycosidic linkages have been identified and their primary structures elucidated.^16^ Serotype III is the most prevalent among GBS strains causing neonatal infection, and the potential of its capsular PS to act as an immunogen is well known.^17^

The development of an effective GBS vaccine that can be used to stimulate the production of protective antibodies in women that can be transplacentally transferred to their babies, still represents the most promising approach to provide protection against GBS neonatal infections,^18^ and PS glycoconjugates of different serotypes are under clinical evaluation. However, the minimal structural epitopes are still unknown for most of the PS variants.

As demonstrated through molecular dynamics simulations and NMR studies,^19^ GBS PSIII tends to form extended helical structures, with a pitch composed of more than four RUs, stabilized by the presence of charged sialic acid residues.^20^ In contrast, the structurally related *S. pneumoniae* type 14 PS, which differs from PSIII by the absence of a sialic acid residue (Neu5Ac) in the lateral chain, results in a more disordered structure.^21^

Enzymatically hydrolyzed PSIII fragments were shown to bind with high affinity to specific monoclonal antibodies (mAbs) in a length‐dependent manner.20b These results were rationalized by the formation of a conformational epitope located on an extended segment of the GBS PSIII involved in antibody recognition. Long polysaccharide portions were also shown to be needed to develop an efficacious vaccine.^22^ Although syntheses of GBSIII related glycans have been reported,^23^ considering the available data, a synthetic carbohydrate based conjugate vaccine against GBS PSIII has been deemed extremely challenging to attain.^24^

**Scheme 1:**
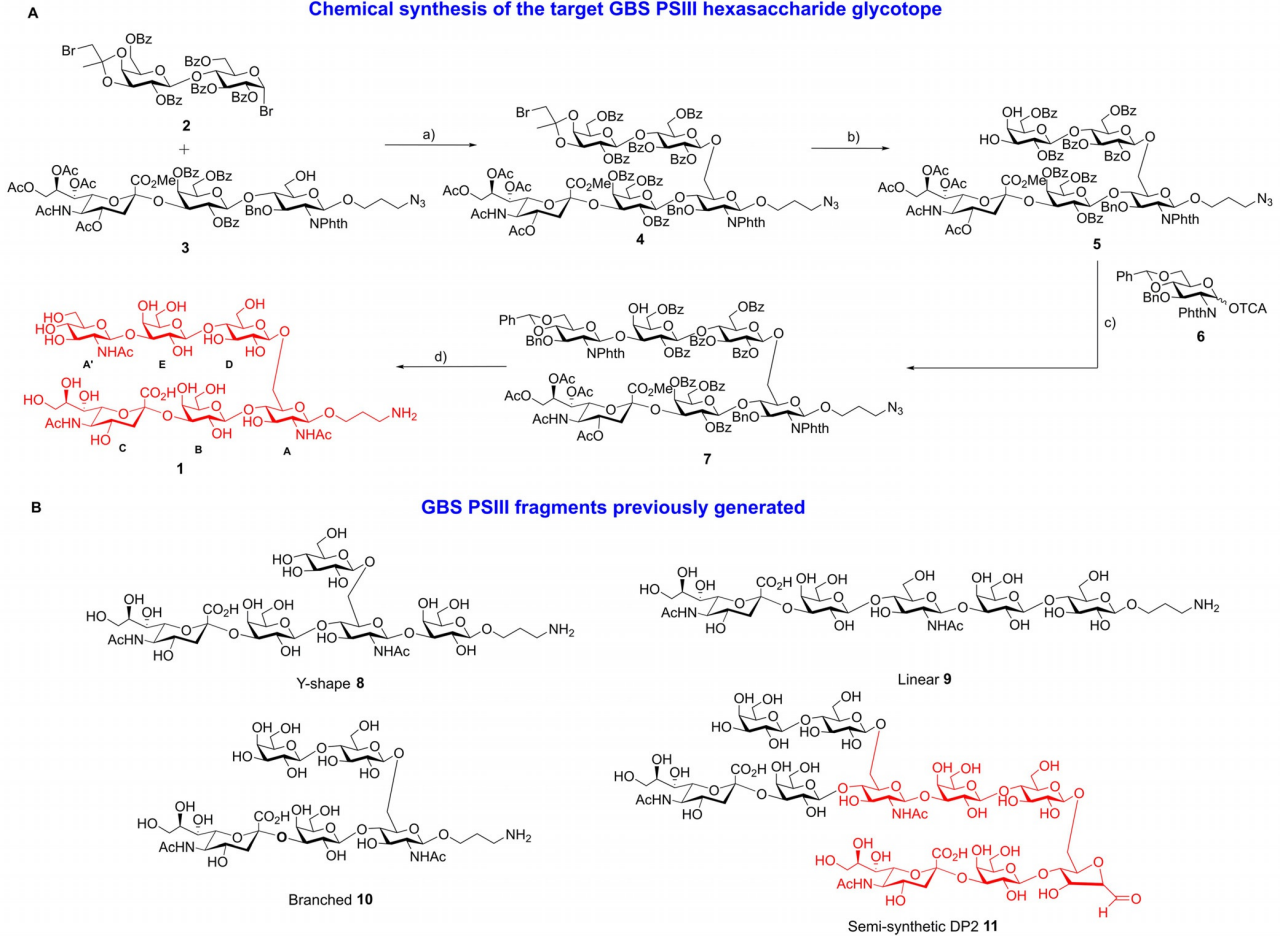
A) Reactions leading to the target hexasaccharide **1**: a) AgOTf, anhydrous dichloromethane, −10 °C, 65 % b) 9:1 TFA/H_2_O, 80 % c) TMSOTf, anhydrous dichloromethane, −30 °C, 69 % d) LiI, Py, 120 °C; H_2_NCH_2_CH_2_NH_2_, EtOH, 90 °C; Ac_2_O‐Py; NaOMe, MeOH; H_2_, Pd‐C, 33 %. B) Structures of GBS PSIII synthetic repeating unit frameshifts and semisynthetic DP2 previously reported.^22^, ^25^

**Figure 1:**
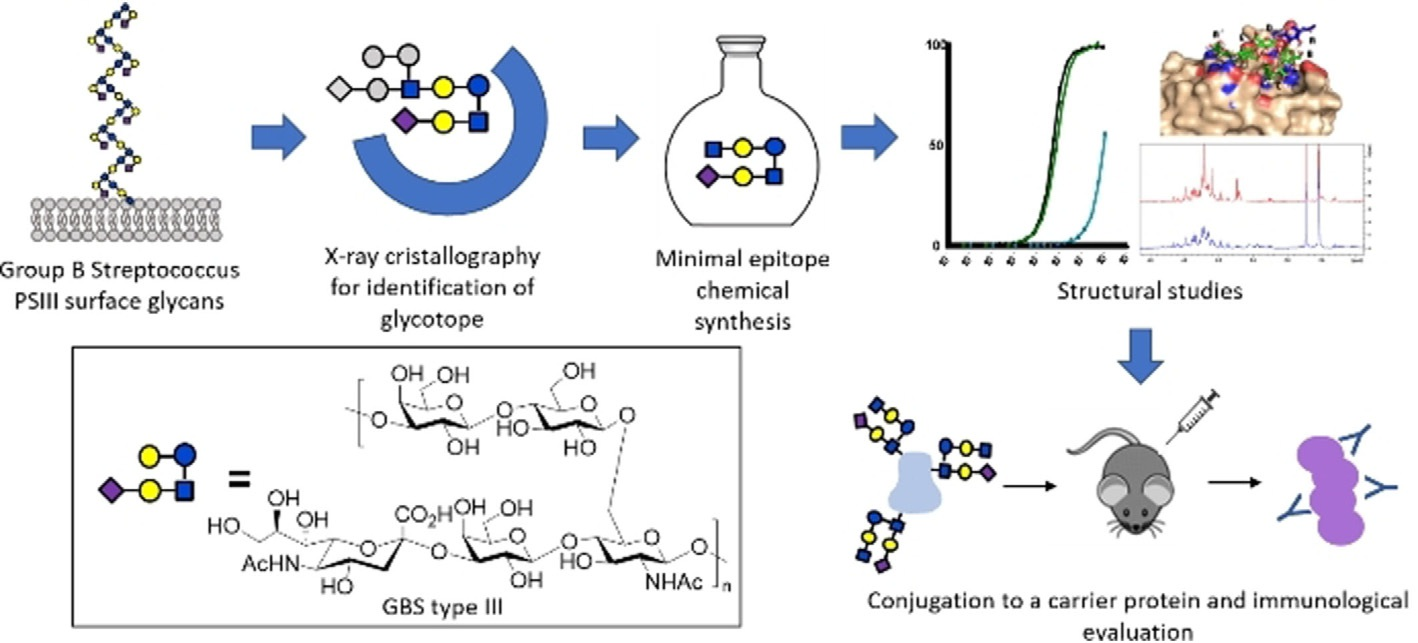
Approach followed for the structure‐guided design of a synthetic carbohydrate based vaccine against Group B *Streptococcus* type III.

Recently we synthesized the three different frameshifts of GBSIII PS^25^ repeating units and, along with semisynthetic fragments obtained by depolymerization^22^ (Scheme 1), we unraveled the molecular details of the interaction with a protective anti‐PSIII mAb. Data generated by combining SPR, STD‐NMR and X‐ray crystallography of a decasaccharide fragment composed of two repeating units (DP2, Scheme 1) complexed with the mAb showed that this fragment contained the PSIII portion necessary for antibody recognition. Particularly, the binding area was sialic acid‐dependent and involved five sugar residues spanning two repeating units.^26^ We subsequently showed that, in contrast to previous reports, a conjugate prepared from the dimer obtained by depolymerization was sufficient to elicit functional antibodies when a substantial number (11) of sugar moieties was incorporated onto the protein.^27^ Based on this evidence, in this work we have designed and synthesized a new hexasaccharide from GBSIII PS representing the minimal antigenic portion containing all the moieties involved in the binding to a protective mAb. By integrating STD NMR experiments and Molecular Dynamics (MD) simulations we demonstrate that this defined oligosaccharide is engaged with the functional mAb through interactions superimposable to those occurring for the recognition of the previously studied dimer fragment. After conjugation to a carrier protein, the hexasaccharide also proved to be the minimal immunogenic polysaccharide epitope. This is the first evidence of an X‐ray crystallography guided design of a synthetic antigen fragment that is also an effective immunogenic epitope for glycoconjugate vaccine development (Figure 1).

## Results and Discussion

### Hexasaccharide fragment synthesis

Hexasaccharide **1**, containing all sugar moieties directly involved in the interaction with a functional mAb binding pocket, was synthesized as described in Scheme 1. Notably, the GlcNAc‐**A**, that in the crystallized semisynthetic dimer was replaced by a furanose residue generated by the utilized depolymerization method, was not involved in the binding, and so it was the optimal residue to be equipped with a linker for conjugation, without epitope alteration.

Therefore, the trisaccharide acceptor **3**,^25^ was glycosylated with the known lactose bromide donor **2** having a participating ester at position 2 of the Glc unit, and an orthogonal isopropylidene protection at the 3,4‐hydroxyls of the Gal residue.^28^ After acid mediated isopropylidene removal, the pentasaccharide **5** was subjected to a regioselective glycosylation at Gal C‐3 with donor **6**, according to a recent protocol we have developed,^29^ to give the protected hexasaccharide **7**. Deprotection was carried out through sequential reactions: 1) hydrolysis of the methyl ester with lithium iodide in pyridine; 2) *N*‐phthalimide removal with ethylenediamine followed by acetylation of the generated amine; 3) methanolysis of the acyl esters and 4) Pd‐charcoal catalyzed hydrogenation to obtain the target glycan **1**.

### Immunochemical and structural analysis of the hexasaccharide

Preliminary analyses were conducted to compare the hexasaccharide fragment **1** with the previously studied semisynthetic DP2 fragment obtained through CPS depolymerization^26^ and a synthesized branched RU pentasaccharide **10** shown in Scheme 1.^25^

**Figure 2:**
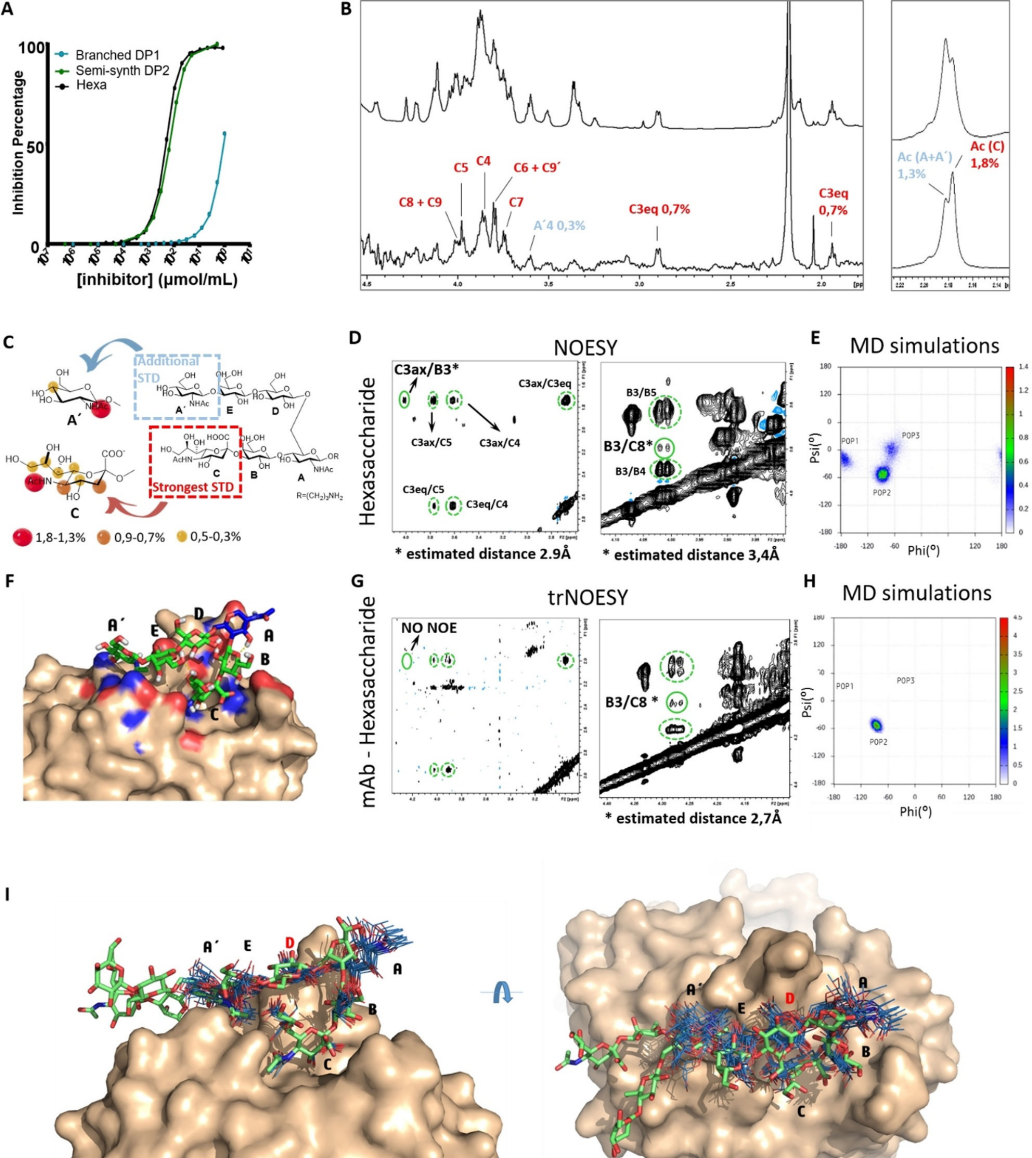
A) Competitive SPR of the binding between the rabbit Fab and CPSIII fragments; B) ^1^H‐STD‐NMR experiment: off‐resonance (top) and STD (below) spectra for the hexasaccharide **1** interacting with mAb, with a zoom on *N*‐acetyl signals on the right. Proton positions receiving strongest saturation after protein irradiation (STD effect) are indicated in red, and isolated proton signals with weaker STD are in blue. C) STD‐NMR interpretation: mapping of the hexasaccharide protons more closely interacting with the mAb. D) NOESY spectrum (free hexasaccharide, 800 MHz) on different regions, showing key interresidual NOE contacts with green circles. Green dashed circles indicate intraresidual NOEs. E) Conformational energy map from MD simulations for the free hexasaccharide **1**. F) Model for the hexasaccharide **1**‐rabbit Fab complex from MD simulation. G) trNOESY spectrum (600 MHz) of the hexasaccharide **1**‐Fab complex on different regions. H) Conformational energy map from MD simulations for hexasaccharide **1** complexed to rabbit Fab. I) Superimposition of different MD frames of the hexasaccharide **1** (in blue) complexed with rabbit Fab (wheat surface) and with the semisynthetic DP2 (in green) from X‐ray (PDB 5m63).

To ascertain the capacity of the newly synthesized hexasaccharide **1** to cover the formerly identified paratope, the compound was first tested by competitive SPR as an inhibitor of the binding of the soluble Fab fragment to a human serum albumin (HSA)‐PSIII conjugate immobilized on the chip (Figure 2 A). The hexasaccharide showed an affinity two orders of magnitude higher than the branched pentasaccharide repeating unit **10** (Scheme 1) and fully mimicked the PSIII DP2. This result confirmed that the upstream GlcNAc‐A′ residue is key for the mAb interaction. The furanoside end terminal residue replacing the downstream GlcNAc‐A in the crystallized glycan dimer, is not hosted in the binding pocket. To gain further details on the sugar residues involved in mAb recognition, ^1^H‐STD‐NMR experiments of the hexasaccharide in complex with the mAb were carried out. The STD spectrum with aromatic protein irradiation (Figure 2 B) showed clear STD signals for specific ligand protons. Remarkably, in spite of the severe signal overlap in the 3.7–4.0 ppm region, all the protons of the Neu5Ac‐C residue could be clearly identified, standing out from the rest of the protons. Additionally, the isolated H3‐C and the signals corresponding to the Me protons of the acetyl groups (one at lower field for GlcNAc‐A′ and GlcNAc‐A residues, and another one at higher field for Neu5Ac‐C) showed an significant STD effect, whereas this effect was weaker for H4‐A. As previously reported, the single branched RU pentasaccharide (Scheme 1) did not exhibit any STD effect for the acetamide of the downstream GlcNAc‐A, but only for that of the Neu5Ac residue (see the Supporting Information, Figure S1).^26^ Therefore, in the STD spectrum of hexasaccharide **1**, the STD effect observed for the Me signal corresponding to both residues GlcNAc‐A′ and GlcNAc‐A, would most likely arise from the GlcNAc‐A′ residue and not from GlcNAc‐A. Thus, despite being far from the hexasaccharide structure, both Ac groups of the GlcNAc‐A′ and Neu5Ac‐C residues, showed a high STD effect, being stronger for the latter compared to the former. These data are in line with the published X‐ray structure, in which the end terminal furanoside residue (equivalent to GlcNAc‐A in the hexasaccharide **1**) was not engaged in interactions to the mAb^26^ and the Neu5Ac‐C residue sited in a binding pocket flanked by aromatic residues, whereas the distal GlcNAc‐A′ was further hooked up by the mAb in a more solvent‐exposed pocket.

Further NMR spectroscopic analysis and MD simulations were carried out to compare the conformational preferences of the hexasaccharide in its free and bound forms (Figure 2 D and H). In the free state, the NOESY spectrum of the hexasaccharide was in the zero‐NOE region at 600 MHz (see the Supporting Information, Figure S2), but showed negative NOEs at 800 MHz. In the presence of the mAb, the NOESY spectrum of the hexasaccharide at 600 MHz showed strong negative NOEs, indicating that they were transferred NOE, arising from the mAb‐bound state of the hexasaccharide. With respect to interglycosidic flexibility, special attention was paid to the linkage Neu5Acα2‐3Gal.

A conformational ensemble involving three conformations was present for this flexible linkage in the free state (Figure 2 D): POP1, where φ/ψ values were ca. 180 °/−20 °, and POP2 and POP3, very close in the conformational energy map, with φ/ψ values ca. −90 °/−50 °, and −65 °/−10 °, respectively. Inter‐residual distances were estimated by using the NOE intensity cross peaks at 800 MHz. Experimentally (Figure 2 E), the strong H3ax Neu5Ac(C)–H3 Gal(B) NOE yielded an estimated distance of 2.9 Å. Since this distance is 2.5, 3.6 and 4.2 Å for POP1, POP2 and POP3 in the conformational models, respectively, this suggested the existence of a conformational equilibrium in solution.

A similar analysis was performed for the H8 Neu5Ac(C)–H3 Gal(B) NOE, which yielded an estimated distance of 3.4 Å. This distance was ca. 4.1 Å in POP1 and 2.6–2.7 Å for POP2 and POP3, corroborating the intrinsic flexibility around this linkage.

Interestingly, the situation was different in the transferred trNOESY spectrum (Figure 2 G). In this case, the only observable NOEs for H3ax of Neu5Ac‐C were the intraresidual ones with H4 and H5, while the inter‐residue contacts were absent. In contrast, the NOE between H3eqNeu5Ac‐C‐H4Gal(B) was more intense in the trNOESY than in the NOESY, yielding an estimated distance for the bound conformation of 2.7 Å.

These data clearly demonstrated the conformational selection process occurring around this linkage upon binding. In particular, in the bound conformation, H3 Gal would be oriented towards the glycerol chain of the Neu5Ac residue, such as in POP2 and POP3, whereas POP1 would not exist.

A MD simulation of the complex between the mAb and hexasaccharide **1** was performed by using the POP2 conformation as starting geometry around the Neu5Ac‐C‐α2‐3‐Gal‐B linkage. The result of the MD simulation is depicted in Figure 2 H, which also shows the superimposition of different frames of the ligand bound to the mAb. As shown, the hexasaccharide complexed with the mAb populates exclusively the POP2 region with a minimum centered at −83 °/−51 °. Remarkably, this value is in perfect agreement with the X‐ray crystallographic structure of the fragment DP2 complexed with the mAb (PDB 5m63), for which the φ/ψ values around Neu5Acα2‐3Gal are −80 °/−16 °. Along the MD simulation, the Neu5Ac‐C residue establishes different intermolecular hydrogen bonds with polar amino acid chains of the protein. In particular, the carboxylate group at C1, the carbonyl moiety of NHCOMe at C5, and the hydroxyl group at C4. On the other hand, the Me group of the NHCOMe is buried in a mAb groove and stacked against the aromatic rings of Y52 and Y115 mAb amino acids of the light chain. Interestingly, the carbonyl group of the NHCOMe of the distal GlcNAc A′ residue also establishes hydrogen bonds with N55 and R66 of the light chain, while the Me group is stacked against Y126 of the heavy chain (Figure 2 F).

The hexasaccharide–mAb complex obtained by NMR spectroscopy and MD analysis was then compared to the X‐ray crystallography‐based complex of DP2 with the Fab already reported (PDB 5m63).^26^ The analysis of the superimposed geometries (Figure 2 I) permitted similar global geometries to be deduced for both complexes. The only difference involves the orientation of the glucose D (Glc‐D) residue (highlighted in red in Figure 2 G), for which the pyranose ring is turned 180 ° with respect to that found in the PDB. Indeed, while the Gal‐E‐Glc‐D and Glc‐D‐GlcNAc‐A linkages adopt unusual non‐exoanomeric and eclipsed conformations (−16 °/−118 ° and −47 °/78 °, respectively) in the DP2 complex, they now display the standard *exo*‐anomeric/*syn* and *exo*‐anomeric/anti geometries, respectively (40 °/20 ° and 30 °/−160 °) for the hexasaccharide complex.

Overall, the combination of ^1^H‐STD‐NMR experiments, NOE analysis of the ligand bound vs. unbound states, and MD simulations of the complex, confirmed a good overlap between the binding mode of new the synthetic hexasaccharide **1** and the previously studied longer DP2 dimer. Importantly, while the newly inserted GlcNAc‐A′ residue is engaged in interactions, the downstream end GlcNA‐A is not and protrudes outside the binding pocket.

Recent in silico conformational studies confirmed that a CPSIII dimer adopts a conformation similar to that adopted in the “zig‐zag” (rather than helical) polymer.^21^ A representative structure obtained from an MD simulation of a helix‐forming polysaccharide fragment composed of ten repeating units was superimposed with the short hexasaccharide structure bound to the mAb (see the Supporting Information, Figure S3). The superimposition highlighted that the conformation of the small minimal interacting epitope fitted the 3D structure acquired by the polysaccharide. This corroborates the conclusion that the hexasaccharide *per se* possesses a well‐defined conformation that allows similar presentation of the key epitope and the polysaccharide. The conformational selection process that involves the Neu5Ac moiety takes place without major energy penalty, which is readily overcome by the new sugar‐antibody intermolecular contacts described above.

### Glycoconjugate synthesis

**Figure 3:**
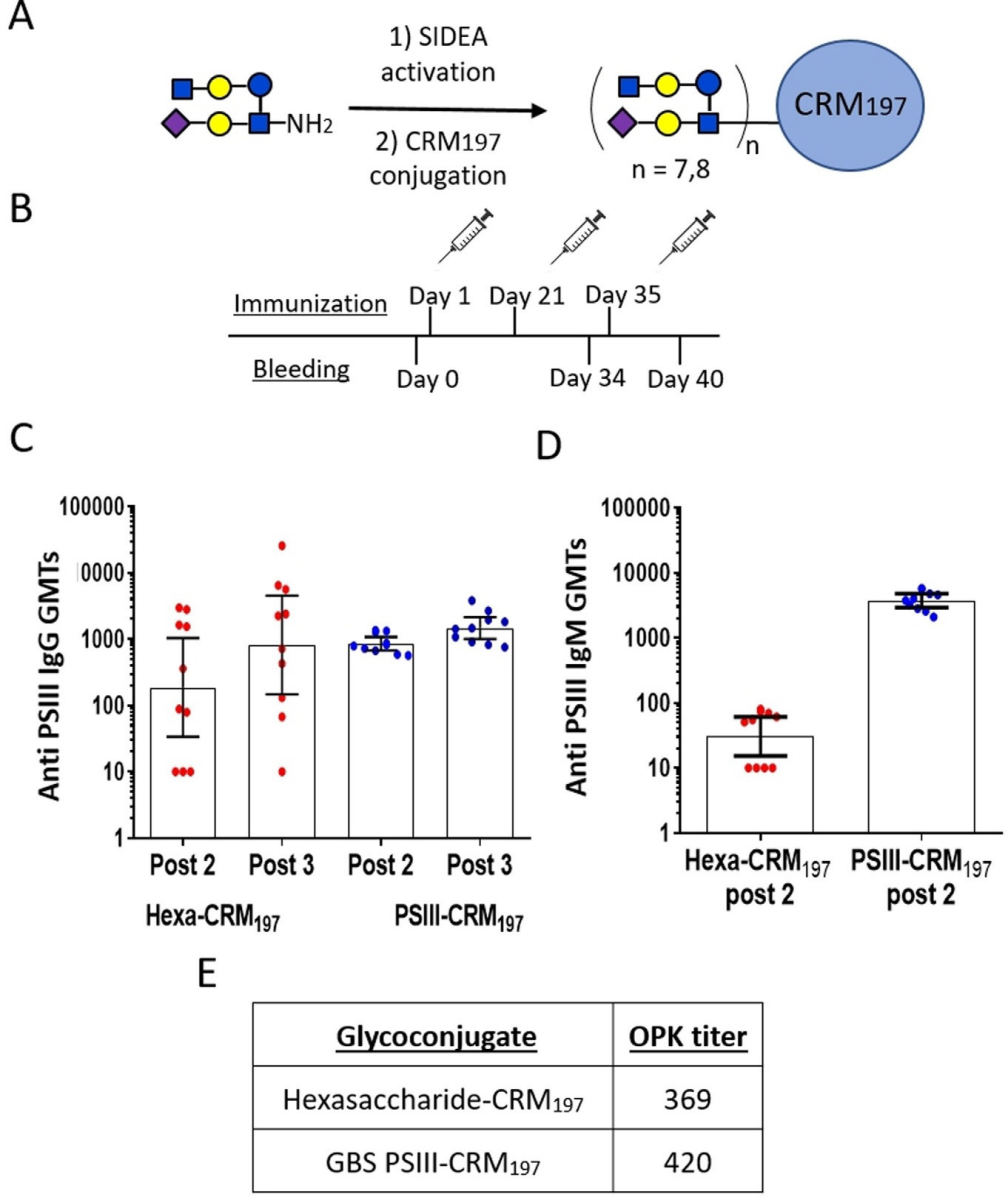
Immunogenicity of the synthetic hexasaccharide conjugate. A) Scheme of hexasaccharide conjugation to CRM_197_. B) Mouse immunization schedule. C) APSIII IgG Geometric Mean titers (GMTs, 95 % CI) after the second and third vaccine injection with CRM_197_‐hexasaccharide and the CRM_197_‐PSIII control. Dots indicate individual mice. Statistical analysis was performed with Kruskal–Wallis and Dunn multiple comparisons test. D) Anti PSIII IgM Geometric Mean Titers after second immunization elicited by CRM_197_‐hexasaccharide and the control CRM_197_‐PSIII. E) OPKA titers measured after the third immunization.

After establishing that hexasaccharide **1** covers the minimal antigenic determinant of PSIII, an immunization study with the hexasaccharide conjugated to the well‐known carrier protein CRM_197_ was envisaged. To this end, the amino‐propyl linker of the hexasaccharide was activated by treatment with an excess of di‐*N*‐hydroxysuccinimidyl adipate and the isolated half ester was incubated with the carrier protein CRM_197_ in sodium phosphate buffer at pH 7.2 with 50:1 saccharide/protein molar ratio. Based on our previous observation that the level of sugar incorporation is crucial to achieve a strong immunogenicity with short GBS PSIII fragments, we optimized the conjugation conditions to reach a high glycosylation level.^27^ The obtained glycoconjugate was purified by filtration and characterized by microBCA for the protein content, while the degree of carbohydrate incorporation was assessed by quantification of the Gal moiety through high‐performance anion‐exchange chromatography coupled with pulsed amperometric detection (HPAEC‐PAD). It was determined that an average of 7.8 glycan moieties were coupled to each CRM_197_ molecule (Figure 3 A).

### Immunological evaluation

We have previously reported the synthesis of GBS glycoconjugates based on the three synthetic pentasaccharide frameshifts **8**–**10** of the type III repeating unit.^25^ Here, in an initial mouse immunization protocol, these conjugates were tested in comparison to a PSIII‐CRM_197_. None of the conjugates elicited specific IgG towards the natural PSIII (see the Supporting Information, Figure S3), except for the branched frameshift (Scheme 1) that, conjugated with a high glycosylation degree (23 sugar moieties/protein), resulted in marginal antibody responses in some of the animals.

Having in hand the conjugated hexasaccharide designed on the basis of the X‐ray DP2‐mAb complex, a second immunization study was undertaken to test its capability of eliciting functional antibodies able to mediate opsonophagocytic killing (OPKA) of GBS strains expressing the type III PS. BALB/c female mice were immunized with three intraperitoneal injections of the synthetic hexasaccharide‐ or the native PSIII‐CRM_197_ conjugates at a saccharide dose of 1.0 μg, formulated with alum hydroxide (Figure 3 B).

PSIII‐specific IgG and IgM induced by the glycoconjugates after the second and third immunization were evaluated by ELISA. As shown in Figure 3 C, IgG levels induced by the novel conjugated synthetic structure did not statistically differ from those elicited by the PSIII conjugate, similarly to what was previously observed with the conjugated semisynthetic dimer fragment.^27^ Of note, the conjugated hexasaccharide elicited a more dispersed immune response than the PSIII control. As recently described, saccharide length may have an impact on T‐cell dependent responses.^30^ Interestingly, the PSIII‐CRM_197_ induced statistically significant (2 log) higher IgM levels than the conjugated hexasaccharide, suggesting that the long PS conjugate might have a higher avidity for specific B‐cell receptors, leading to maturation of plasma B cells and higher IgM production, in concomitance with a T cell dependent response. Conversely, the synthetic oligosaccharide elicited primarily IgG responses.

Antibody functional activity was assessed by an in vitro OPK assay, which is a well‐established technique to mimic the in vivo process of GBS killing after bacterial opsonization by effector cells in the presence of complement and specific antibodies. In agreement with ELISA analysis, OPKA of pooled sera after three doses of hexa‐CRM_197_ vaccine indicated comparable functional antibody titers to those of the PSIII‐CRM_197_ control group (Figure 3 E).

Taken together, these data clearly indicate that hexasaccharide **1**, containing the essential sugar residues needed to antibody binding, was also the minimal immunogenic PSIII epitope. The obtained results also suggested that multivalent presentation of short synthetic glycans could mimic multiple epitope presentation as in the natural polysaccharide.^31^ Finally, the upstream GlcNAc‐A′ of the hexasaccharide was proven to be crucial not only for antibody recognition but also for immunogenicity.

## Conclusions

The GBS capsular polysaccharide is known as a primary virulence factor and an optimal target for vaccine development. The type III is the most frequent of the ten existing serotypes among neonatal invasive infection strains. For many years, the PSIII has been considered the prototype of a length‐dependent conformational glyco‐epitope, but we have recently shown how a fragment composed of two repeating units is sufficient to interact with a functional anti PSIII rabbit monoclonal antibody, covering its binding pocket. In particular, by combining X‐ray crystallography, SPR and STD‐NMR spectroscopic results of a PSIII dimer complexed with a protective mAb, we demonstrated the existence of a sialic acid‐dependent antigenic determinant that is fully contained within six sugars deriving from both the PSIII backbone and the disaccharide arm. This structure was subsequently proven to trigger a functional response in mice after conjugation to a carrier protein, demonstrating that it contained the minimal immunogenic epitope.^27^ Based on these findings, here we designed and synthesized a hexasaccharide structure to be tested for its immunogenicity. Synthetic carbohydrates would offer advantages for glycoconjugate vaccines in terms of absence of potential bacterial contaminations, high reproducibility and improved in‐process analytical control.^32^

The antigenic nature of the assembled hexasaccharide was confirmed by competitive SPR spectroscopy, through which the short glycan was proven to compete with the full‐length PSIII for binding to a protective mAb, comparably to the dimer previously obtained by PS depolymerization. Furthermore, STD‐NMR spectroscopy highlighted how the acetamides of the sialic acid and of the upstream glucosamine A′ were in close proximity to the mAb binding site, and therefore involved in the binding. Molecular modeling simulations corroborated these observations by showing the conformational preferences of the hexasaccharide in the binding pocket of the mAb. Superimposition of the complex mAb–DP2 and the simulated mAb–hexasaccharide showed that both are very similar and share most of the contact points. The conformation adopted by the hexasaccharide in the binding with antibodies was dictated by both the presentation of the polysaccharide and stabilizing interactions with the antibody.^21^ Overall, these data indicate that the hexasaccharide is the minimal antigenic determinant recognized by a protective anti PSIII mAb.

Conjugation of the hexasaccharide to the well‐known carrier protein CRM_197_ allowed its immunogenicity to be evaluated in vivo. The obtained glycoconjugate elicited similar levels of functional antibodies to the native polysaccharide conjugate.^33^ Although previous reports suggested GBSIII is an extremely complex epitope and pointed out the necessity of using long polysaccharide portions to contain immunogenic epitopes, our structure‐guided approach sheds further light on this paradigm by demonstrating that a small synthetic glycan can be designed to merely contain the carbohydrate moieties key for a robust immunogenicity. This study also underpins how structural glycobiology can aid deciphering the sugar glycocode and simplify the design of vaccines based on complex bacterial polysaccharides. In addition, the results support the notion that any complex polysaccharides can be potentially replaced by small well‐defined glycan epitopes obtainable through chemical or chemoenzymatic assembly.^34^

## Experimental Section

Animal studies were authorized by the Italian Ministry Of Health and were undertaken in accordance with the regulations of the Directive 2010/63/EU. Full experimental details can be found in the Supporting Information.

## Conflict of interest

D.O., L.D.B., F.C., P.R.M.H., F.A., M.M.R., F.B., I.M., and R.A. are employees of GSK groups companies. L.D.B., F.C., F.B., I.M., and R.A. are inventors of patents related to the topic. F.B., I.M., and R.A. are owners of GSK stocks.

## Supporting information

As a service to our authors and readers, this journal provides supporting information supplied by the authors. Such materials are peer reviewed and may be re‐organized for online delivery, but are not copy‐edited or typeset. Technical support issues arising from supporting information (other than missing files) should be addressed to the authors.

Supplementary
