# Supplementary material for: Structure‐Guided Design of a Group B Streptococcus Type III Synthetic Glycan–Conjugate Vaccine
Source: Chemistry. 2020 Apr 1;26(31):7018–25. doi: 10.1002/chem.202000284 (PMC7317837; doi:10.1002/chem.202000284)
Supplement: Supplementary file 1 — Supplementary [file CHEM-26-7018-s001.pdf]

Chemistry–A European Journal

Supporting Information

Structure-Guided Design of a Group B *Streptococcus* Type III Synthetic Glycan–Conjugate Vaccine

Davide Oldrini,[a] Linda del Bino,[a] Ana Arda,[b] Filippo Carboni,[a] Pedro Henriques,[a] Francesca Angiolini,[a] Jon I. Quintana,[b] Ilaria Calloni,[b] Maria R. Romano,[a] Francesco Berti,[a] Jesus Jimenez-Barbero,[b, c, d] Immaculada Margarit,[a] and Roberto Adamo*[a]

Table of Contents

Material and methods..2

Procedures for the synthesis of hexasaccharide **1**……………………………………………2

Conformational studies, conjugation and immunogenicity……………………………………7

STD NMR of branched pentasaccharide RU…………………………………………………..11

TrNOESY NMR……………………………………………………………………………….11

Immunogenicity of GBS PSIII pentasaccharide repeating unit frameshifts…………………...12

MD simulations of GBS PSIII……………..……………………………………………………..13

References…………………………………………………………………………………...18

Material and methods

General Methods

Reactions were monitored by thin-layer chromatography (TLC) on Silica Gel 60 F254 (Sigma Aldrich); after exam under UV light, compounds were visualized by heating with 10% (v/v) ethanolic H_2SO_4. In the work up procedures, organic solutions were washed with the amounts of the indicated aqueous solutions, then dried with anhydrous Na_2SO_4, and concentrated under reduced pressure at 30–50ºC on a water bath. Column chromatography was performed on Silica Gel 60 (Sigma Aldrich, 0.040–0.063 nm) or using pre-packed silica cartridges RediSep (Teledyne-Isco, 0.040–0.063 nm) or Biotage SNAP Ultra (Biotage, silica 0.050 nm). Unless otherwise specified, a gradient 0 → 100% of the elution mixture was applied in a Combiflash Rf (Teledyne-Isco) or Biotage Isolera instrument. Solvent mixtures less polar than those used for TLC were used at the onset of separation. ^{1}H NMR spectra were measured at 400 MHz and 298 K with a Bruker AvanceIII 400 spectrometer; δH values are reported in ppm, relative to internal Me_4Si (δH = 0.00, $CDCl_3$); solvent peak for D_2O was calibrated at 4.79 ppm. ^{13}C NMR spectra were measured at 100 MHz and 298 K with a Bruker AvanceIII 400 spectrometer; δC values are reported in ppm relative to the signal of $CDCl_3$ (δC = 77.0, $CDCl_3$). Assignments of NMR signals were made by homonuclear and heteronuclear 2-dimensional correlation spectroscopy, run with the software supplied with the spectrometer. Assignment of ^{13}C NMR spectra of some compounds was aided by comparison with spectra of related substances reported previously from this laboratory or elsewhere. When reporting assignments of NMR signals, sugar residues in oligosaccharides are indicated with capital letters. Exact masses were measured by electron spray ionization cut-off spectroscopy, using a Q-Tof micro Macromass (Waters) instrument. Structures of these compounds follow unequivocally from the mode of synthesis, NMR data and m/z values found in their mass spectra.

Procedures for the synthesis of hexasaccharide 1

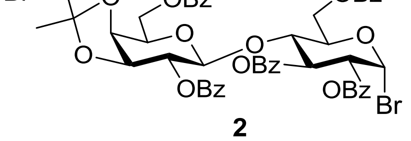

2

[2,6-Di-O-benzoyl-3,4-O-(1-bromomethylethylidene)-β-D-galactopyranosyl]-(1→4)-2,3,6-tri-O-benzoyl-α-D-glucopyranosyl bromide (2). Compound **2** was synthesised as previously reported; ^{1}H NMR and ^{13}C NMR are in line with ones reported in literature[1].

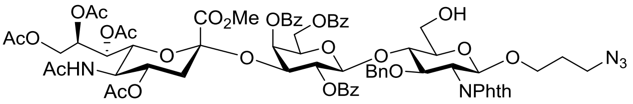

3

3-Azidopropyl 2,4,6-tri-*O*-benzoyl-3-*O*-(methyl 4,7,8,9-tetra-*O*-acetyl-5-*N*-acetamido-3,5-dideoxy-D-glycero-α-D-galacto-non-2-ulopyranosylonate)-β-D-galactopyranosyl)-(1→4)-3-*O*-benzyl-2-deoxy-2-phthalimido-β-D-glucopyranoside (3). Compound **3** was synthesised as previously reported; ^{1}H NMR and ^{13}C NMR are in line with ones reported in literature.[2]

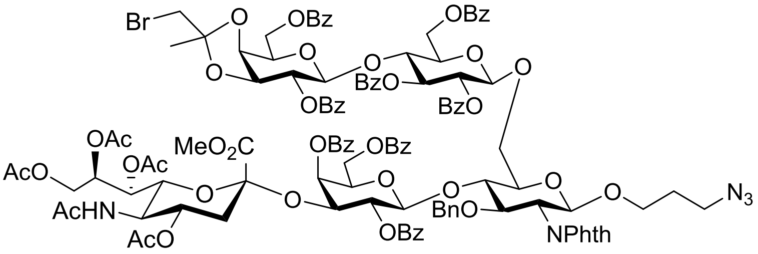

4

3-Azidopropyl [2,6-di-O-benzoyl-3,4-O-(1-bromomethylethylidene)-β-D-galactopyranosyl-(1→4)-2,3,6-tri-O-benzoyl-β-D-glucopyranosyl-(1→6)]-[2,4,6-tri-O-benzoyl-3-O-(methyl 4,7,8,9-tetra-O-acetyl-5-N-acetamido-3,5-dideoxy-D-glycero-α-D-galacto-non-2-ulopyranosylonate)-β-D-galactopyranosyl-(1→4)]-3-*O*-benzyl-2-deoxy-2-phthalimido-β-D-glucopyranoside (4). A solution of donor **2** (420 mg, 0.60 mmol) and trisaccharide acceptor **3** (330 mg, 0.24 mmol) with activated molecular sieves (4 A, 800 mg) in DCM (8 mL) was stirred for 20 min under nitrogen. AgOTf (77 mg, 0.30 mmol) was added at -40 °C. The reaction mixture was stirred for 10 h at rt, when TLC (7:3 Tol:acetone) showed complete reaction. TEA was added, the solid filter off and the solvent removed at reduced pressure. The crude was purified by flash chromatography (Tol:acetone 8:2) to afford **4** (370 mg, 0.16 mmol) in 65 %yield. $[\alpha]_D^{25}$ = + 42.73° (c 1.4, CHCl3). ESI MS m/z $[M+H]^+$ found 2395.1274; calcd 2395.1439

^{1}H NMR (400 MHz, $CDCl_3$) δ 8.08-6.58 (m, 49H, H-Ar), 5.71 (t, J = 8.90 Hz, 1H, H-8^C), 5.45-5.37 (m, 2H, H-2^B, H-3^D), 5.22-5.14 (m, 3H, H-2^D, H-4^B, H-2^E), 5.07 (dd, J = 2.84, 9.86 Hz, 1H, H-7^C), 4.92 (d, J=10.2, 1H, NH), 4.82-4.68 (m, 5H, H-1^A, H-1^B, H-3^B, H-4^C, C*H*HPh^A), 4.42 (d, J=7.22, 1H, H-1^E), 4.39-4.24 (m, 5H), 4.20- 4.16 (m, 2H, H-1^D, CH*H*Ph^A), 4.05 (dd, J = 5.35, 11.22 Hz, 1H, H-9^C), 3.97-3.86 (m, 6H), 3.73 (m, 6H), 3.63-3.42 (m, 7H, incl. OCH_{2a}), 3.25 (q, J=8.69, 2H, CH_2Br), 3.10-3.05 (m, 1H, OCH_{2b}), 2.91-2.84 (m, 2H, CH_2N_3), 2.77-2.73 (m, 1H, H-5^E), 2.38 (dd, 1H, H-$3^C{}_e$), 2.03, 1.75, 1.71, 1.66, 1,53 (5 x s, 3H each, 5 x CH_3CO), 1.60 [s, 3H, C(CH_3)], 1.54 (m, 1H, H-$3^C{}_a$), 1.37-1.28 (m, 2H, OCH_2CH_2).

^{13}C NMR (101 MHz, $CDCl_3$) δ 170.80-164.89 (13x C=O esters) 134.20-122.37 (m, 49, C-Ar), 101.88 (C-1^D), 100.99 (C-1^B), 100.54 (C-1^E), 97.93 (C-1^A), 96.87, 80.25, 78.18, 78.03, 75.68, 74.97, 74.62, 74.56, 73.39, 72.70, 72.45, 72.72, 72.58, 72.52, 72.43, 71.06, 70.58, 70.41, 69.46, 68.16, 67.54, 67.02, 66.85, 66.18, 63.21, 62.21, 62.76, 62.33, 61.55, 55.76, 53.23, 48.62, 48.01, 37.31 (C-3^C), 37.07, 28.62, 24.58, 23.14, 21.33, 20.75, 20.73, 20.43.

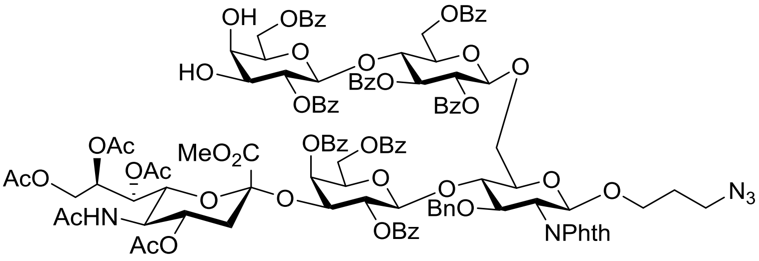

5

3-Azidopropyl [2,6-di-*O*-benzoyl-β-D-galactopyranosyl-(1-4)-2,3,6-tri-*O*-benzoyl-β-Dglucopyranosyl-(1-6)]-[2,4,6-tri-*O*-benzoyl-3-*O*-(methyl 4,7,8,9-tetra-*O*-acetyl-5-*N*-acetamido-3,5-dideoxy-D-glycero-α-D-galacto-non-2-ulopyranosylonate)-β-D-Galactopyranosyl-(1-4)]-3-*O*-benzyl-2-deoxy-2-phthalimido-β-D-glucopyranoside (5). Pentasaccharide **4** (370 mg, 0.16 mmol) was dissolved in a 90% solution of TFA. After 1h rt, TLC (Toluene:Acetone 6:4) showed complete reaction. Reaction was concentrated under reduced pressure and purified via flash chromatography (Tol:acetone 7:3) giving **4** (302 mg, 0.13 mmol) in 83% yield as a white solid.

$[\alpha]_D^{25}$ = + 38.78° (c 1.5, CHCl3). ESI MS m/z $[M+H]^+$ found 2276.9871; calcd 2277.1840.

^1H NMR (400 MHz, CDCl3) δ 8.08-6.58 (m, 49H, H-Ar), 5.69 (t, J = 8.90 Hz, 1H, H-8^C), 5.46-5.38 (m, 2H, H-2^B, H-3^D), 5.26-5.17 (m, 3H, H-2^D, H-2^E, H-4^B), 5.08 (dd, J = 2.36, 9.81 Hz, 1H, H-7^C), 4.98 (d, J = 10.07, 1H, NH), 4.80-4.68 (m, 5H, H-1^B, H-1^A, H-4^C, H-3^B, C*H*HPh), 4.45 (d, J = 7.81, 1H, H-1^E), 4.39-4.32 (m, 2H, H-$9^C{}_a$, H-$6^E{}_a$), 4.26 (d, J = 7.64, 1H), 4.13 (d, J = 12.25, 1H, CH*H*Ph), 3.97-3.85 (m, 9H, incl. H-2^A, H-4^E), 3.72 (m, 6H, incl. H-5^C, H-$9^C{}_b$, $COOCH_3$), 3.64-3.59 (m, 3H), 3.56-3.44 (m, 5H, incl. OCH_{2a}), 3.16-3.10 (m, 1H, OCH_{2b}), 2.91 (q, J = 6.04, 2H), 2.83-2.79 (m, 1H), 2.37 (dd, J = 4.62, 12.79, 1H, H-3^C), 2.01, 1.83, 1.70, 1.67, 1.52, (5 x s, 3H each, 5 x CH_3CO), 1.46 (m, 1H, H-3^C), 1.36 (m, 2H, OCH_2CH_2)

^{13}C NMR (101 MHz, $CDCl_3$) δ 170.0-164.9 (13x C=O esters), 134.0-125.1 (C-Ar), 101.66 (C-1^D), 101.04 (C-1^B), 100.98 (C-1^E), 97.93 (C-1^A), 96.89, 80.15, 74.95, 74.57, 73.69, 72.99, 72.51, 72.39, 71.68, 71.58, 71.45, 70.58, 70.09, 69.48, 68.17, 67.00, 66.86, 66.19, 63.14, 62.91, 61.70, 61.53, 55.74, 53.24, 48.61, 48.02, 46.71, 37.31 (C-3^C), 28.64, 23.13, 21.33, 20.75, 20.72, 20.45

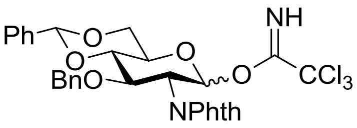

6

3-O-benzyl-4,6-O-benzylidene-2-deoxy-2-phthalimido-D-glucopyranoside-(α,β)-trichloroacetimidate (6). Compound **6** was synthesised as previously reported; ^{1}H NMR and ^{13}C NMR are in line with ones reported in literature[3].

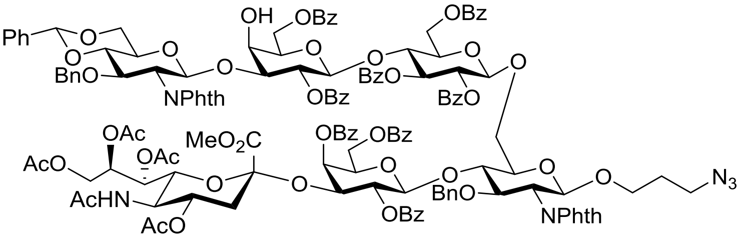

7

3-Azidopropyl [3-*O*-benzyl-4,6-O-benzyliden-2-deoxy-2-phthalimido-β-D-glucopyranosyl-(1-3)-2,6-di-*O*-benzoyl-β-D-galactopyranosyl-(1-4)-2,3,6-tri-*O*-benzoyl-β-D-glucopyranosyl-(1-6)]-[2,4,6-tri-*O*-benzoyl-3-*O*-(methyl 4,7,8,9-tetra-*O*-acetyl-5-*N*-acetamido-3,5-dideoxy-D-glycero-α-D-galacto-non-2-ulopyranosylonate)-β-D-galactopyranosyl-(1-4)]-3-*O*-benzyl-2-deoxy-2-phthalimido-β-D-glucopyranoside (7). A solution of glucosamine donor **6** (68 mg, 0.11 mmol) and acceptor **5** (190 mg, 0.08 mmol) was stirred for 20 min in dry DCM with activated molecular sieves 4Å under nitrogen. TfOH was then added at -25°C and the reaction was stirred for 1h at 0°C. After that, TLC (Toluene:Acetone 6:4) showed complete reaction, so the mixture was quenched with TEA, the solid was filtered of and the crude was purified with flash chromatography. Hexasaccharide **7** (164mg, 0.06 mmol) was obtained has a white amorphous solid in 69% yield.

$[\alpha]_D^{25}$ = + 36.31° (c 0.32, CHCl3). ESI MS m/z $[M+H]^+$ found 2745.8362; calcd 2745.6693.

^{1}H NMR (400 MHz, $CDCl_3$) δ 8.04-6.61 (m, 63H, H-Ar), 5.73 (t, J = 9.09, 1H, H-8^C), 5.55 (s, 1H, C*H*Ph), 5.46 (t, J = 8.46, 1H, H-2^B), 5.30-5.10 (m, 6H, H-1^F, H-3^D, H-2^D, H-2^E, H-4^B, H-7C), 4.91 (d, J = 10.04, 1H, NH), 4.79-4.68 (m, 6H, H-1^B, H-1^A, C*H*HPh^A, C*H*HPh^F), 4.36-4.22 (m, 6H, incl. H-1^E), 4.12-4.03 (3H), 3.98-3.87 (m, 7H, incl. H-1^D), 3.83-3.67 (m, 11H), 3.62-3.47 (m, 8H, OCH_{2a}), 3.14-3.08 (m, 1H, OCH_{2b}), 2.94-2.90 (m, 2H, CH_2N_3), 2.49-2.40 (m, 2H, H-3^C, H-5^E), 2.06, 1.91, 1.77, 1.67, 1.59 (5 x s, 3H each, 5 x CH_3CO), 1.58 (m, 1H, H-3^C), 1.37 (m, 2H, $CH_2CH_2N_3$).

^{13}C NMR (101 MHz, $CDCl_3$) δ 170.80-164.24 (13 x C=O esters), 138.00-125.11 (C-Ar), 102.04 (C-1^D), 101.36 (*C*HPh), 101.01 (C-1^B), 100.88 (C-1^E), 99.98 (C-1^F), 97.88 (C-1^A), 82.66, 80.59, 80.53, 78.12, 75.57. 75.01, 74.43, 74.18, 74.03, 72.45, 72.33, 72.06, 71.67, 71.54, 71.42, 71.33, 70.82, 70.64,

70.47, 69.53, 98.53, 68.40, 68.14, 67.07, 66.80, 66.23, 66.11, 63.25, 62.71, 62.46, 61.50, 55.73, 55.50, 53.25, 48.57, 48.03, 37.36 (C-3[C]), 28.59, 23.13, 21.49, 21.32, 20.77, 20.34.

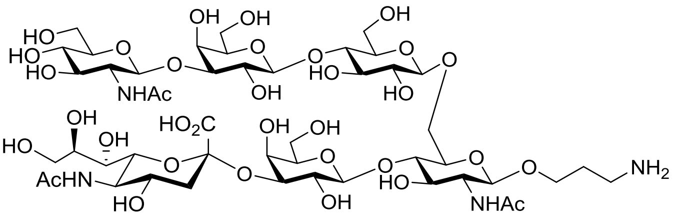

1

3-Aminoopropyl 3-O-(5-N-acetamido-3,5-dideoxy-D-glycero-α-D-galacto-non-2-ulopyranosyl)-β-D-galactopyranosyl)-(1→4)-O-[(2-acetamido-2-deoxy-β-D-glucopyranosyl)-(1→3)-(β-D-galactopyranosyl)-(1→4)-O-(β-D-glucopyranosyl)-(1→6)]-O-2-acetamido-2-deoxy-β-D-glucopyranoside (1). A mixture of protected hexasaccharide **7** (0.1 mmol) and LiI (3 mmol) in pyridine (5 mL) was heated for 24 h at 120 °C. The reaction mixture was concentrated under vacuum, and the residue was purified by silica gel column chromatography (gradient 2 % MeOH in DCM) to afford the demethylated product. This material was dissolved in ethanol (4 mL), and ethylenediamine (400 μL) was added. After being stirred for 16 h at 90 °C, the reaction mixture was then concentrated in vacuo, and the residue was coevaporated from Toluene (2 x 10 mL) and EtOH (2 x 5 mL). The crude mixture was re-dissolved in pyridine (5 mL), and acetic anhydride (5 mL) was added. After being stirred for 16 h at room temperature, the reaction mixture was concentrated under reduced pressure and the residue was purified by silica gel column chromatography (gradient 10 % MeOH in DCM). The residue was dissolved in MeOH and MeONa was added until pH = 13.

After 48 h the reaction was neutralized and the solvent removed under vacuum and the crude was purified with C18 5g column (gradient 20% MeOH in H_2O). The residue was finally dissolved in MeOH and Pd/C (1 : 1 w/w in respect to the sugar) was added. The reaction mixture was stirred under pressure of H_2 (3 bar) for 72 h. Then, the catalyst was filtered off and the filtrate concentrated under reduced pressure. The reaction mixture was purified by G-10 size-exclusion column chromatography using water for elution. Fractions containing the sugar were quantified by sialic acid assay and freeze-dried to afford the deprotected oligosaccharide **1** as an amorphous powder (31 % yield).
$[\alpha]_D^{25}$ = + 1.56° (c 0.81, H_2O). ESI MS m/z $[M+Na]^+$ found 1281,4932; calcd 1281,4610.
^{1}H NMR (400 MHz, D_2O) δ 4.68 (d, J = 8.4 Hz, 1H, H.1^F), 4.60 (d, J= 7.9 Hz, 1H, H-1^B), 4.53 (d, J= 8.49 Hz, 1H, H-1^D), 4.50 (d, J= 8.63 Hz, H1, H-1^A), 4.43 (d, J= 7.77 Hz, 1H, H-1^E), 4.30, (d, J= 10.5 Hz, 1H, H-6$^A{}_a$), 4.14 (d, J= 2.7 Hz, 1H, H-4^E), 4.08, (dd, J= 2.7, 9.8 Hz, 1H, H-3^B), 3.99-3.80 (8H), 3.79-3.54 (22H), 3.46-3.44 (2H), 3.34 (t, J= 8.2 Hz, 1H, H-2^E), 3.20 (t, J= 8.6 Hz, 2H, CH_2N_3), 2.75,

(dd, J= 4.6, 12.4 Hz, 1H, H-3^{C}_{eq}), 2.03 (s, 6H, 2 x CH_3CO), 2.02 (s, 3H, CH_3CO), 1.97 (m, 2H, $CH_2CH_2N_3$), 1.80 (t, J= 12.4 Hz, 1H, H-3^{C}_{ax})

^{13}C NMR (101 MHz, D_2O) δ 102.91 (C-1^{E}), 102.81 (C-1^{F}), 102.42 (C-1^{D}), 102.12 (C-1^{B}), 101.33 (C-1^{A}), 81.87, 78.20, 77.25, 75.60, 75.00, 74.85, 74.58, 74.24, 73.50, 75.44, 72.91, 72.62, 72.08, 71.75, 69.98, 69.93, 69.33, 68.33, 67.98, 67.51, 67.30, 62.56, 61.05, 60.93, 60.42, 59.99, 55.60, 55.01, 51.63, 49.09, 47.37, 39.59 (C-3^{C}), 23.52, 22.11, 22.01.

Conformational studies, conjugation and immunogenicity

SPR Fab binding inhibition. Inhibition assay were performed by SPR using a BIACORE X100 system. HSA-PSIII Glycoconjugate was immobilized on CM5 sensor chips (Biacore) using the amine coupling kit supplied by the manufacturer (Biacore). Immobilizations were conducted in 10-20mM sodium acetate (pH 4-5) at sugar concentrations of 2-4μg/mL. The immobilized surface density was ~50 resonance units in each instance. Binding analysis was performed with samples of Fab at a fixed concentration pre-incubated with PSIII or its fragments serially diluted (2x) starting from a concentration of 2mg/mL. Measurements were conducted in 10 mM HEPES (pH 7.2), 150mM NaCl, 3mM EDTA, 0.005% Tween20 at 25°C and at a flow rate of 45μL/min. Following mAb or Fab binding, conjugate surfaces were regenerated with 3.5M $MgCl_2$ and a contact time of 120s. Sensorgram data were analyzed using BIAevaluation software (Biacore).

NMR Sample preparation . mAb were exchanged in the working buffer (Tris d-11 50 mM in D2O at pH 8.0 +/- 0.1) through 2 mL Zeba Spin desalting column saturated with 3 cycles of working buffer, mAb were finally eluted at the same starting concentration of 1 mg/ml. The 100% of recovery was assessed through microBCA spectrophotometric assay. Hexasaccharide fragment was desalted, quantified, dried and then dissolved in the working buffer too. mAb final concentration in the NMR tube was of 4 μM mAb:ligand ratio was set at 50:1 mol/mol for the DP1. For the STD of the hexasaccharide **1**, 11 μM of GBSIII antibody was used in 20 mM PBS buffer in D_2O and pH 7 with 100:1 mol/mol ratio for the hexasaccharide.

STD NMR experiments. NMR experiments were carried out on a Bruker 600 MHz NMR instrument equipped with a QCI cooled probe at controlled temperature (± 0.1 K). Data acquisition and processing were performed using TOPSPIN 3.5 software, respectively. Suppression of water signal was achieved by excitation sculpting (2 msec selective square pulse). STD-NMR experiments were acquired with 72 scans over 72 accumulations and spectral width of 9600 Hz (16 ppm) at 303 K; a saturation transfer of 0,5/1/1,5/2/4 sec. were applied to enhance the saturation transfer effect,

irradiating at a frequency of 8.0 and -1.0 ppm (2 different spectra recorded for each sample). No differences were observed in the STD spectra irradiating at 8.0 or -1.0 ppm (4800 and -600 Hz respectively) for all samples, confirming that the saturation transfer effect was not irradiation dependent. To avoid pitfalls in the interpretation of STD-NMR spectra, a negative control spectrum was always recorded in absence of mAb (ligand and buffer) at the very same condition (concentration and pH) of the mAb-saccharide samples. Increasing saturation times from 0.5 up to 4.0 sec were applied to avoid overseeing of possible bias in the calculation of STD effects due to the different proton longitudinal relaxation times (T1), as well as the intramolecular spin diffusion within the bound state.

For the hexasaccharide **1**, the STD experiment was performed on a Bruker Advance 800 MHz spectrometer with cryoprobe. The experiment was carried out at 318 K recording 768 scans over 12 accumulations and spectral width of 12000 Hz (15.4 ppm). The protein saturation was achieved by using a Gaussian shape pulse of 50ms, with a total saturation time of 2 sec. at frequencies of 7.2 and 0.4 ppm (5800 and 360 Hz respectively.

Conjugation to CRM197. Triethylamine (3.0 eq) was added to a 9:1 DMSO/water solution of hexasaccharide, followed by di-N-hydroxysuccinimidyl adipate (12 eq). The reaction was stirred for 3 h, then the product was precipitate at 0°C by adding ethyl acetate (9 volumes). The solid was washed 10 times with ethyl acetate (5 volumes each) and lyophilized. The activated sugar was conjugated to CRM197 in sodium phosphate 100 mM at a protein concentration of 20 mg/mL, using the saccharide/protein molar ratio of 50:1.

After incubating overnight, the glycoconjugate was purified by dialysis against 10 mM sodium phosphate buffer pH 7.2 (30 washings) in 30 kDa Vivaspin Turbo (Sartorius) centrifugal concentrators and reconstituted in the same buffer.

Immunogenicity of conjugates in mice. Two groups of ten female BALB/c mice were immunized by intraperitoneal injection of 1 μg in saccharide content of each produced glycoconjugate using alum hydroxide as an adjuvant. CRM-PSIII was used as control. Mice received the vaccines at days 1, 21 and 35. Sera were bled at days 1, 35 and 49.

ELISA analysis using GBS PS III conjugated to human serum albumin (HSA) as coating reagent (IgG). Microtiter plates (96 wells, NUNC, Maxisorp) were coated with 100 μL of 1μg/mL of GBS PSIII conjugated to HSA via the spacer adipic acid dihydrazide in Phosphate Buffered Saline (PBS) pH 7.4. Plates were incubated overnight at 2-8°C, washed three times with PBST (0.05%

Tween-20 in PBS pH 7.4) and saturated with 250 µL/well of PBST-B (2% Bovine Serum Albumin-BSA in PBST) for 90 min at 37°C. The plates were then aspirated to remove the solution. Two-fold serial dilutions of test and standard sera in PBST-B were added to each well. Plates were then incubated at 37°C for 1h, washed with PBST, and then incubated for 90 min at 37°C with anti-mouse IgG-alkaline phosphatase (Sigma) diluted 1:2000 or anti-rabbit IgG-alkaline phosphatase diluted 1:1000 in PBST-B. After washing, the plates were developed with a 4 mg/mL solution of p-Nitrophenyl Phosphate in 1 M diethanolamine pH 9.8, at room temperature for 30 min. After blocking with 7% EDTA, the absorbance was measured using a SPECTRAmax plate reader with wavelength set at 405 nm. IgG concentrations were expressed as relative ELISA Units/mL (EU/mL) and were calculated by interpolating the absorbance values of serial sample dilutions on the standard calibration curve (Reference line method). The murine standard consists of a pool of hyperimmune sera obtained from animals immunized with 3 doses of GBS PS III-CRM conjugate vaccines adjuvanted with Alum.

ELISA analysis using GBS PS III conjugated to human serum albumin (HSA) as coating reagent (IgG). Microtiter plates (96 wells, NUNC, Maxisorp) were coated with 100 µL of 1 µg/mL of GBS PSIII conjugated to HSA via the spacer adipic acid dihydrazide in Phosphate Buffered Saline (PBS) pH 7.4. Plates were incubated overnight at 2-8°C, washed three times with PBST (0.05% Tween-20 in PBS pH 7.4) and saturated with 250 µL/well of PBST-B (2% Bovine Serum Albumin-BSA in PBST) for 90 min at 37°C. The plates were then aspirated to remove the solution. Two-fold serial dilutions of test and standard sera in PBST-B were added to each well. Plates were then incubated at 37°C for 1h, washed with PBST, and then incubated for 90 min at 37°C with anti-mouse IgM-alkaline phosphatase (Sigma) diluted 1:2000. After washing, the plates were developed with a 4 mg/mL solution of p-Nitrophenyl Phosphate (pNPP) in 1 M diethanolamine (DEA) pH 9.8, at room temperature for 30 min. After blocking with 7% EDTA, the absorbance was measured using a SPECTRAmax plate reader with wavelength set at 405 nm. IgG concentrations were expressed as relative ELISA Units/mL (EU/mL) and were calculated by interpolating the absorbance values of serial sample dilutions on the standard calibration curve (Reference line method). The murine standard consists of a pool of hyperimmune sera obtained from animals immunized with 3 doses of GBS PS III-CRM conjugate vaccines adjuvanted with Alum.

Opsonophagocytosis Killing Assay (OPKA). Functional activity of anti-GBS antibodies was estimated by Opsono Phagocytic Killing Assay (OPKA) using differentiated HL-60 cells and strains COH1-III[2]. The percent of killing was calculated as (mean Colony Forming Units at T0 - mean CFU at T60)/(mean CFU at T0). OPK titers were expressed as the reciprocal serum dilution mediating 50%

bacterial killing, estimated through piecewise linear interpolation of the dilution-killing OPK data. The Lower Limit of Detection was 1:30 and the assay coefficient of variation was approximately 30%.

Conformational analysis of the hexasaccharide. Free state. NOESY experiments of hexasaccharide were acquired at 600 MHz (200 and 400 ms mixing time) and at 800 MHz (200 and 600 ms mixing time). 2D-ROESY experiment was also acquired at 600 MHz (200 ms of mixing time).
Bound state. trNOESY experiments were acquired for a sample of 11 µM of mAb and 20 equivalents of hexasaccharide, at 600 MHz (100, 200 and 400 ms mixing times) and at 800 MHz (200 ms mixing times).

MD simulations. The initial geometry of the glycan was built using the carbohydrate builder module available in the GLYCAM web portal (Glycam Biomolecule Builder). The initial pdb coordinates for mAb were derived from the crystal structure Protein Database (PDB) 5m63. The glycan was manually docked into the binding site.
MD simulations of 500 ns were run using Amber 16 with the ff14SB force field parameters for the protein and GLYCAM06j-1 for the glycan. The simulations were prepared in explicit TIP3P water and minimized in two steps before starting the simulation. The analysis of the MD trajectories was carried out using the ccptraj module included in Amber-Tools 16 package.

STD NMR of branched pentasaccharide RU

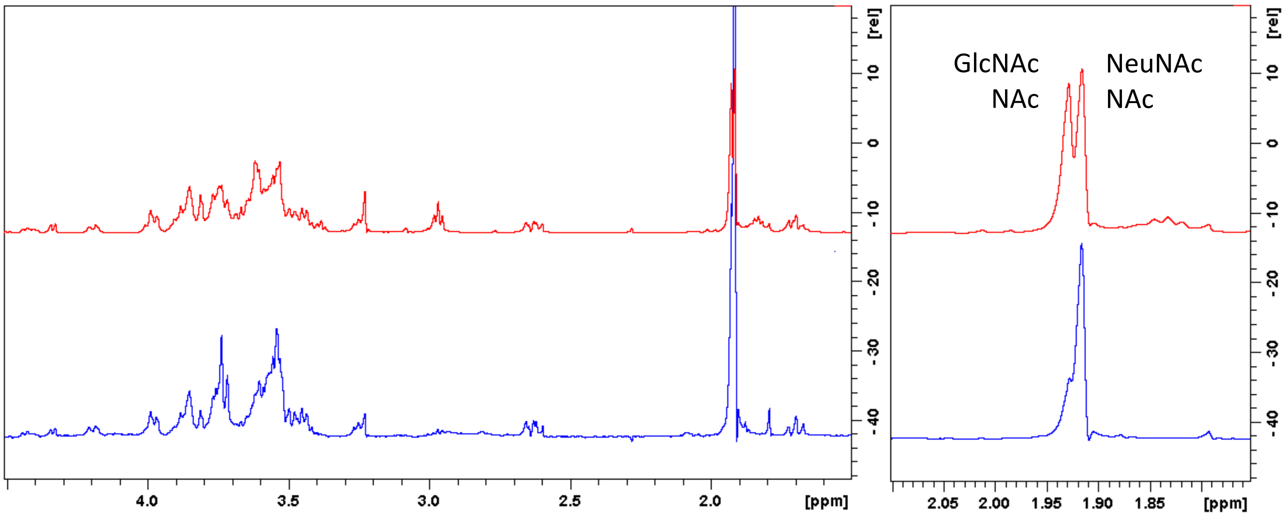

Figure S1. mAb-Branched DP1 STDD-NMR spectrum (bottom) and of 1H NMR spectrum (top) of branched DP1 frameshift. Zoomed spectra shows strong interaction was identified for NeuNAc acetamide, while low interaction with mAb was detected for GlcNac acetamide.[4]

trNOESY NMR

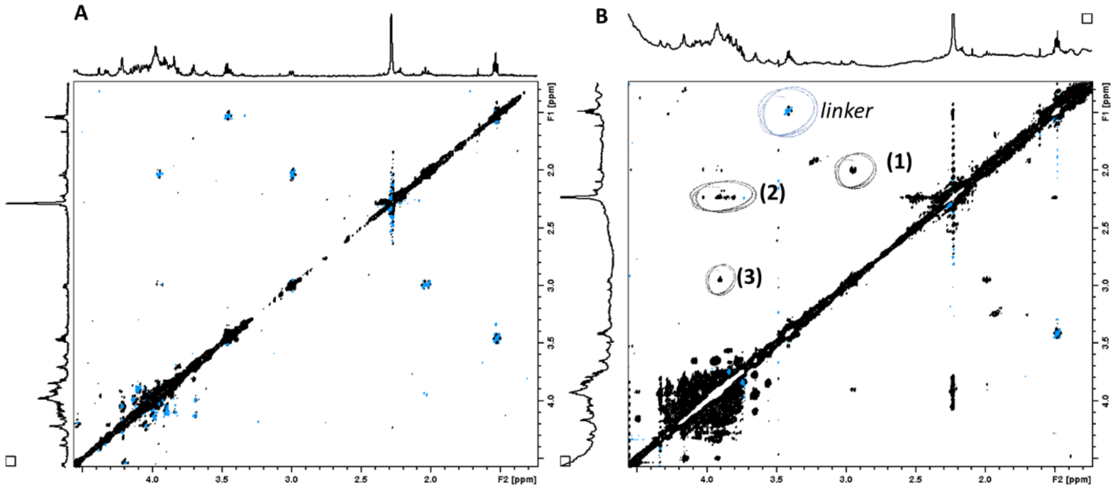

Figure S2. The NOESY spectrum of the hexasaccharide free at 323K, 600MHz and 400ms of mixing time in free (A) and bound (B) state.

MD simulations of GBS PSIII

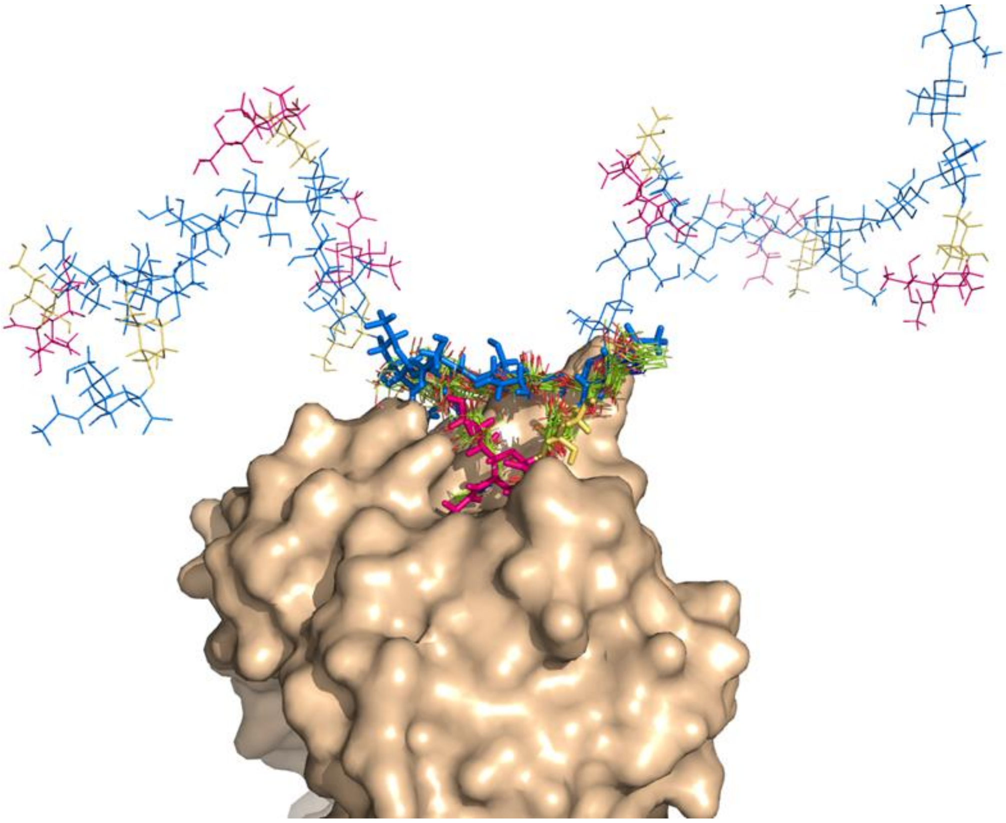

Figure S3: Complex for the hexasaccharide **1** (thin lines in lime, different frames) with the mAb (pdb 5m63) (surface in wheat) according to MD. A representative structure of a helix-forming polysaccharide fragments composed of ten repeating units, obtained from MD is superimposed. The polysaccharide residues at the binding site are represented in thick lines, while the rest of the polysaccharide is in thin lines. Colour code: Neu5Ac and Gal at the branches are magenta and yellow respectively, while residues of the backbone (-3Galβ1-4Glcβ1-6GlcNAcβ1-) are in blue. The superimposition demonstrates that the conformation of this small minimal interacting epitope (hexasaccharide **1**) perfectly fits the helix formed by the polysaccharide.

Immunogenicity of GBS PSIII pentasaccharide repeating unit frameshifts

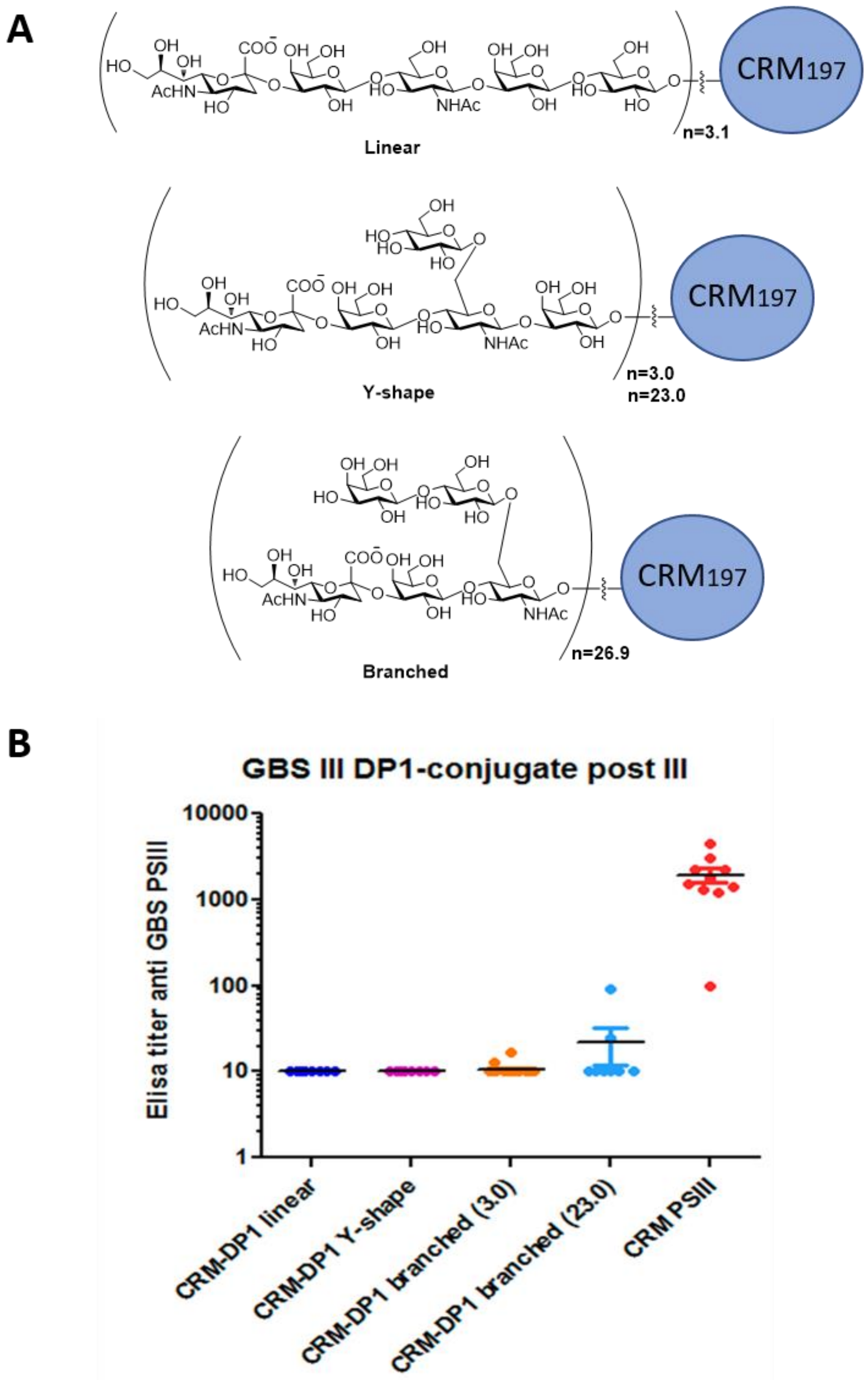

Figure S3: A) Schematic representation of glycoconjugates obtained from different synthetic GBS PSIII RU frameshifts and final saccharide/protein molar ratios. B) Anti-GBS PSIII IgG ELISA titers after third doses of glycoconjugates administered to BALB/c mice at a 0.5 µg carbohydrate dose at days 1, 21 and 35. GBS PSIII was used for ELISA coating. Each dot represents an individual animal. The horizontal bars indicate the geometric mean titer and 95% confidence intervals.

NMR Spectra of synthesized compounds

Compound 4: ^{1}H NMR, CDCl$_3$, 400 MHz

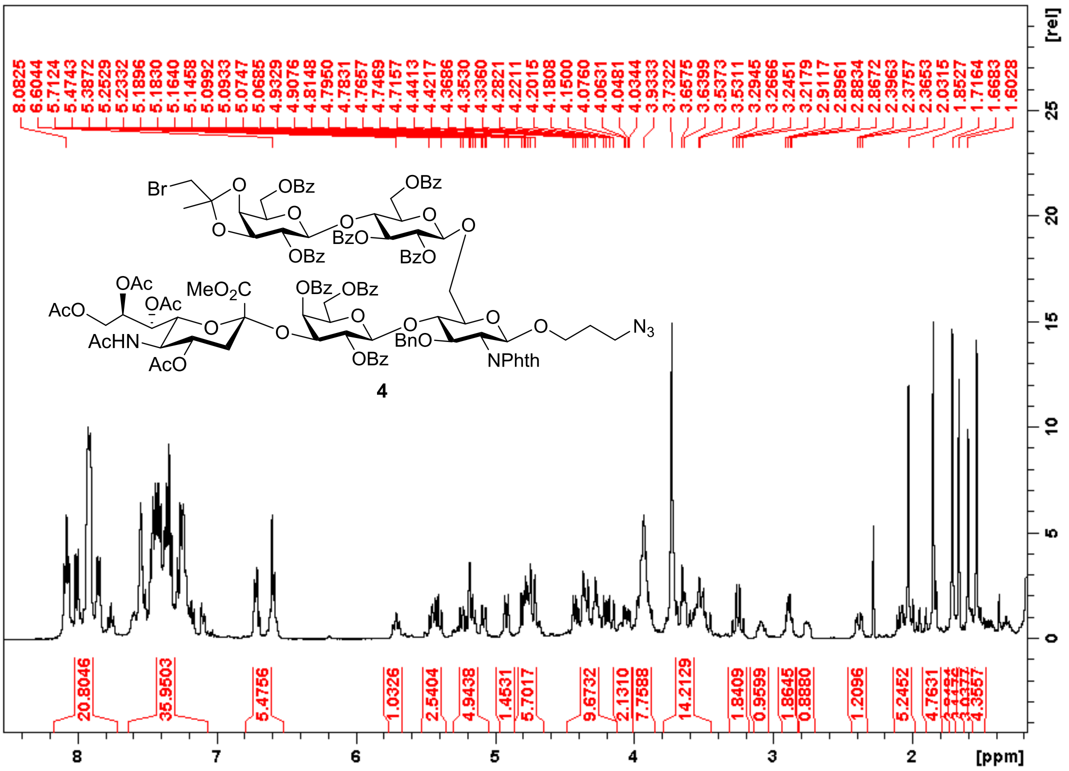

Compound 4: ^{13}C NMR, CDCl$_3$, 101 MHz

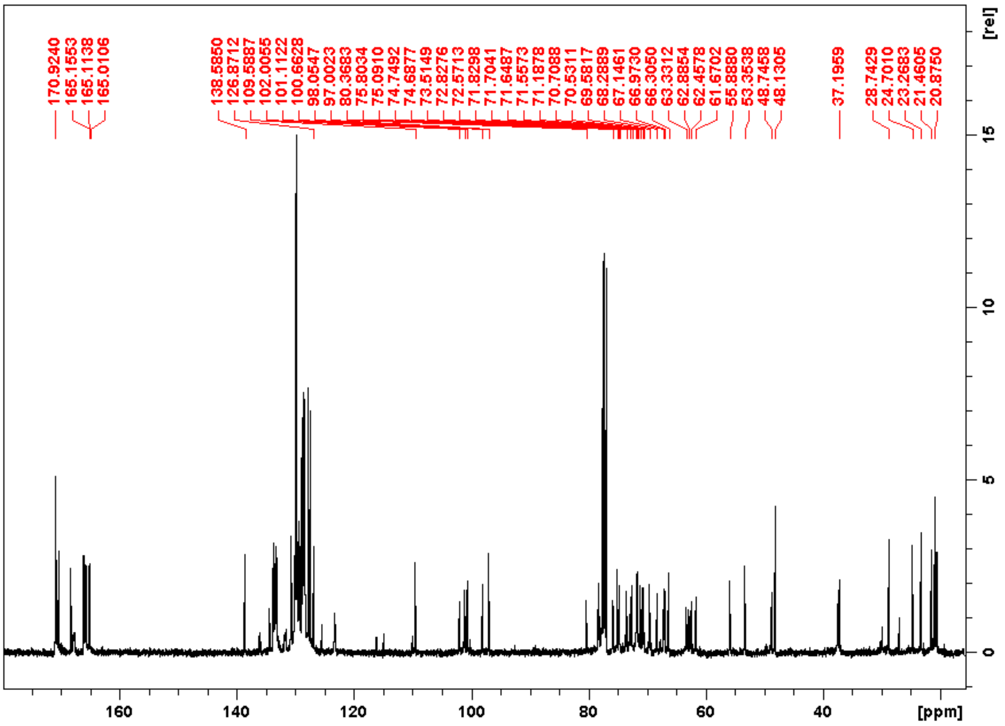

Compound 5: ^{1}H NMR, $CDCl_3$, 400 MHz

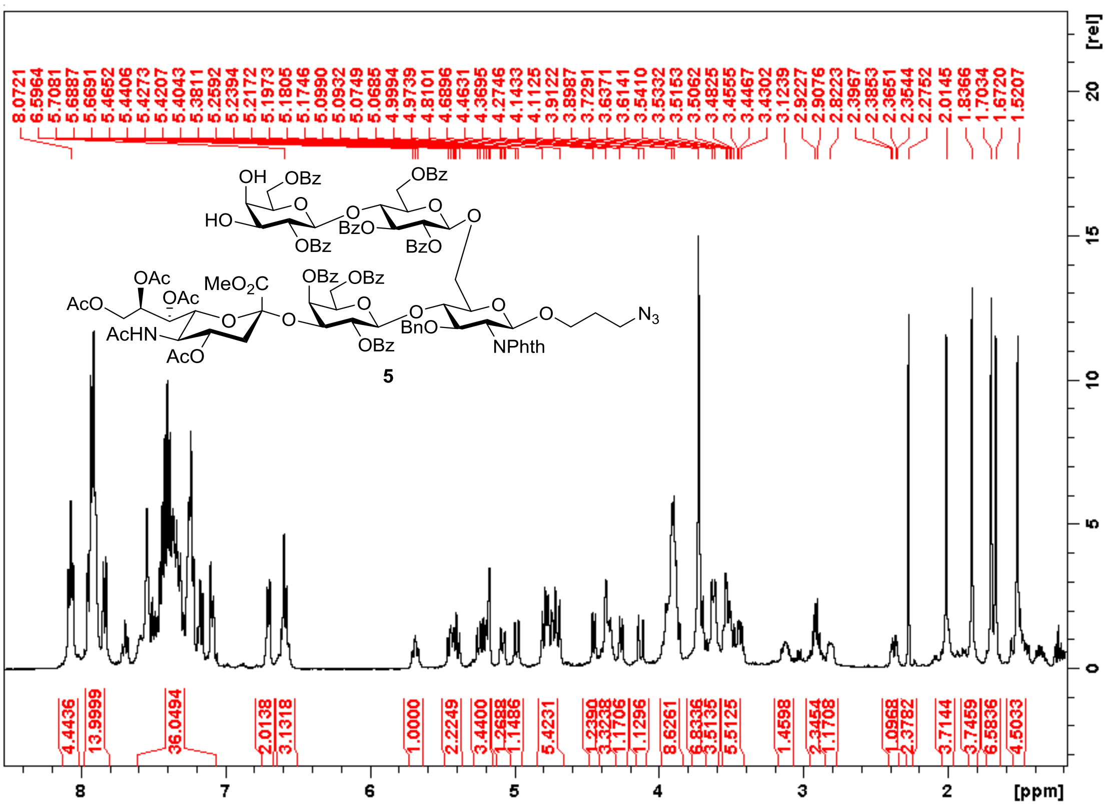

Compound 5: ^{13}C NMR, $CDCl_3$, 101 MHz

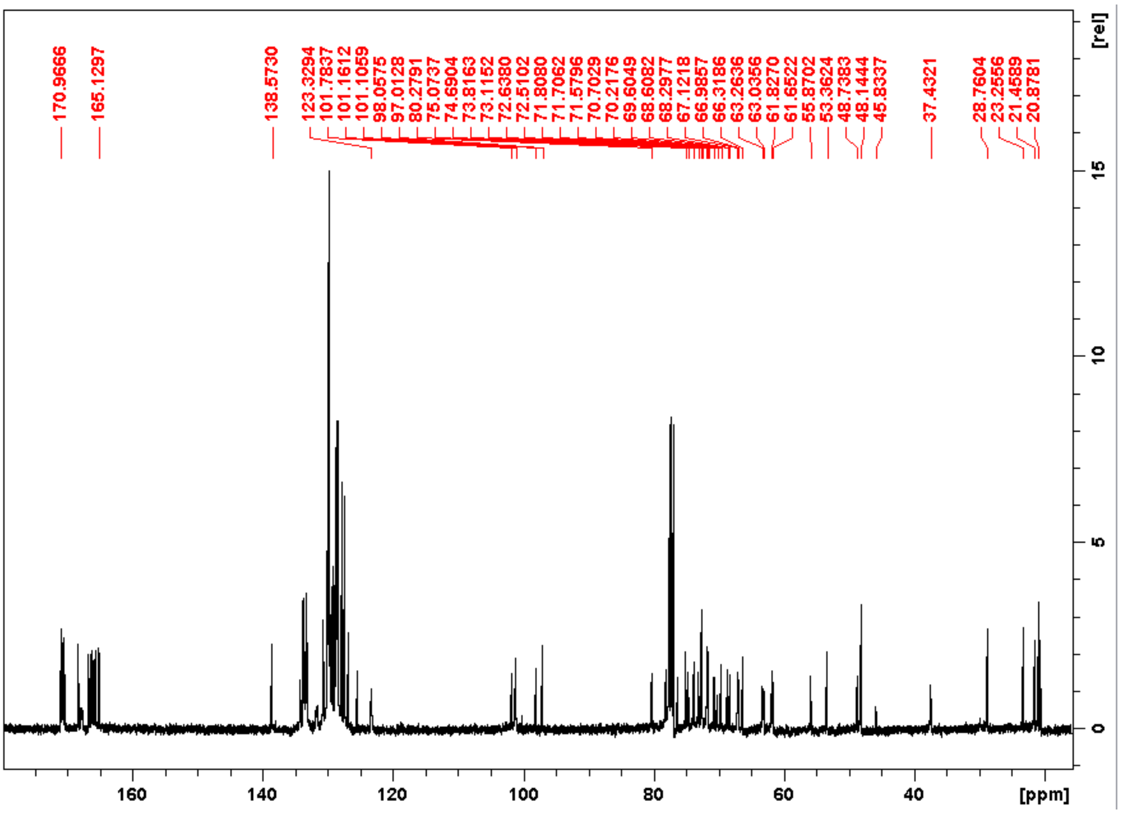

Compound 7: ^{1}H NMR, $CDCl_3$, 400 MHz

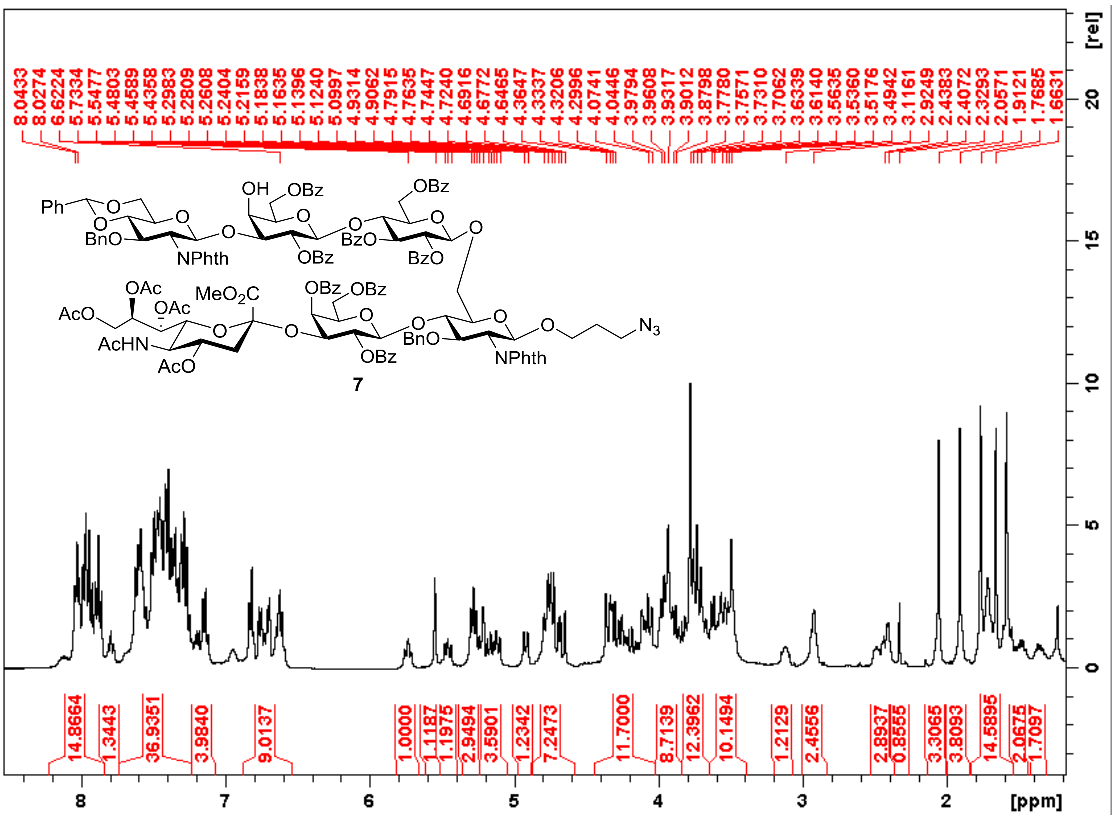

Compound 7: ^{13}C NMR, $CDCl_3$, 101 MHz

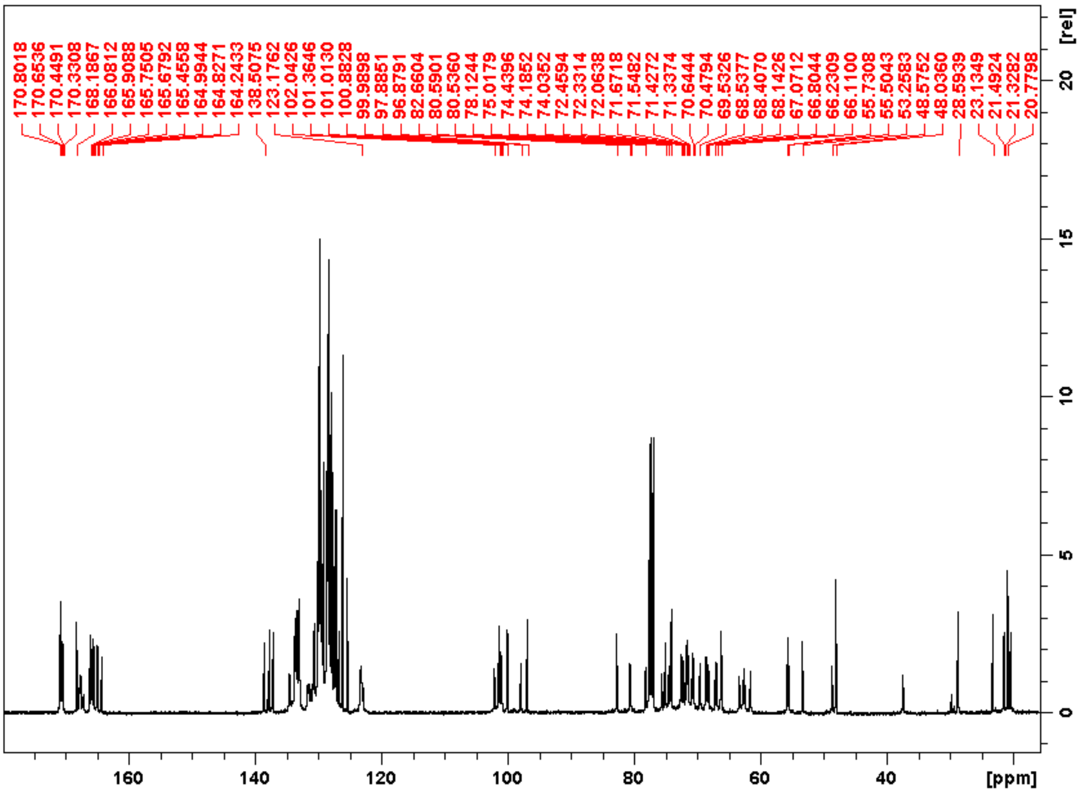

Compound 1: ^{1}H NMR, D_2O, 400 MHz

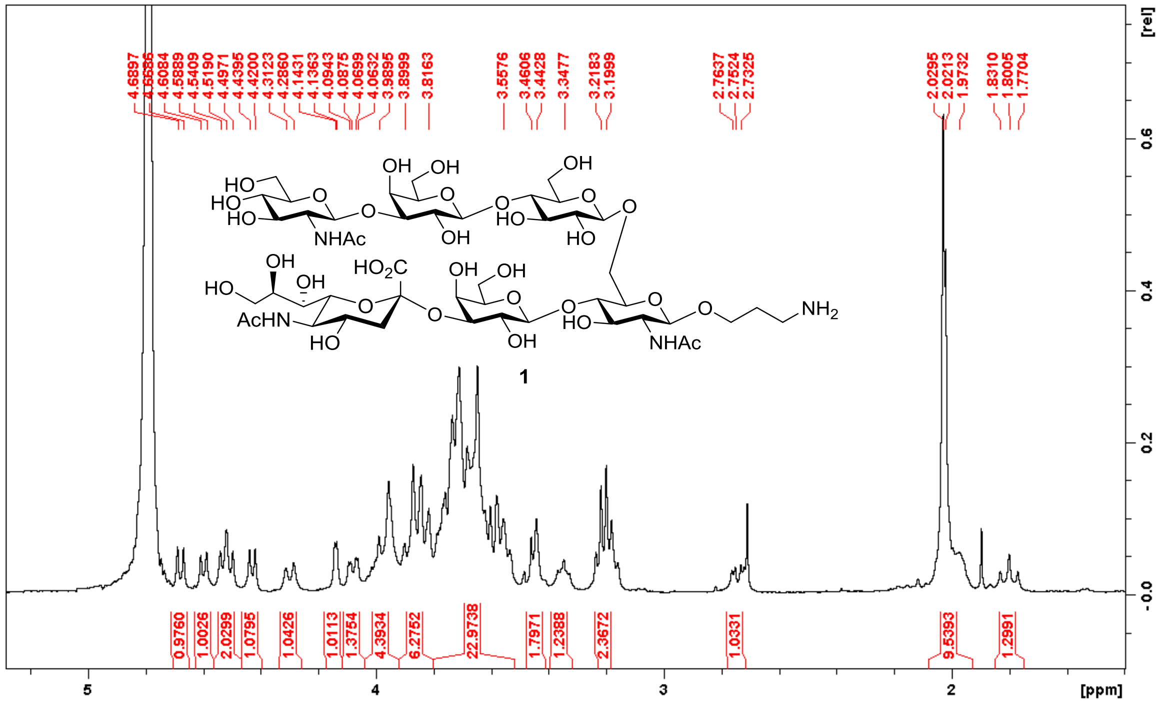

Compound 1: ^{13}C NMR, $CDCl_3$, 101 MHz

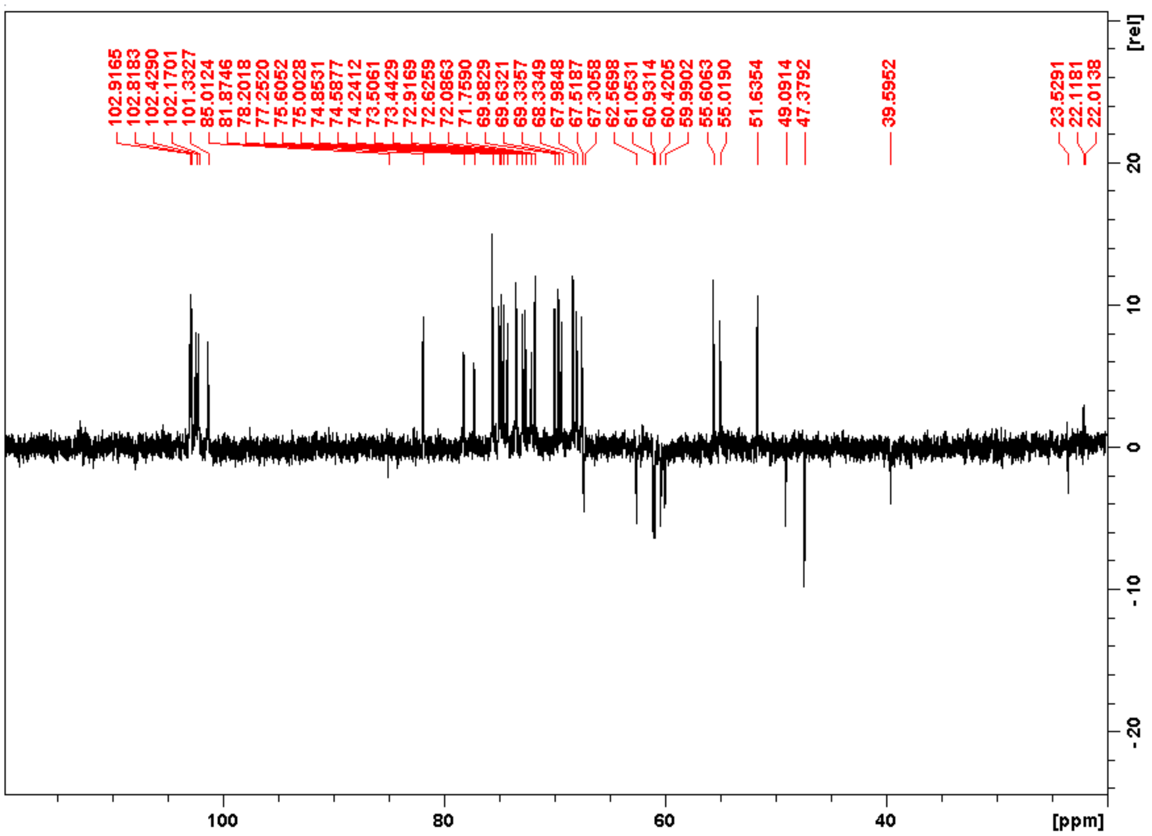

References

1. Sundgren, A.; Lahmann, M.; Oscarson, S., Block Synthesis of Streptococcus pneumoniae Type 14 Capsular Polysaccharide Structures*. *Journal of Carbohydrate Chemistry* **2005,** *24* (4-6), 379-391.
2. Cattaneo, V.; Carboni, F.; Oldrini, D.; De Ricco, R.; Donadio, N.; Margarit, I.; Berti, F.; Adamo, R., Synthesis of Group B Streptococcus type III polysaccharide fragments for evaluation of their interactions with monoclonal antibodies. *Pure Appl. Chem.* **2017,** *89*, 855-875.
3. Del Bino, L.; Calloni, I.; Oldrini, D.; Raso, M. M.; Cuffaro, R.; Arda, A.; Codee, J. D. C.; Jimenez-Barbero, J.; Adamo, R., Regioselective Glycosylation Strategies for the Synthesis of Group Ia and Ib Streptococcus Related Glycans Enable Elucidating Unique Conformations of the Capsular Polysaccharides. *Chemistry* **2019**.
4. Carboni, F.; Adamo, R.; Fabbrini, M.; De Ricco, R.; Cattaneo, V.; Brogioni, B.; Veggi, D.; Pinto, V.; Passalacqua, I.; Oldrini, D.; Rappuoli, R.; Malito, E.; Margarit, I. Y. R.; Berti, F., Structure of a protective epitope of group B Streptococcus type III capsular polysaccharide. *Proc. Natl. Acad. Sci. U. S. A.* **2017,** *114* (19), 5017-5022.
